# Wnt5A modulates integrin expression in a receptor-dependent manner in ovarian cancer cells

**DOI:** 10.1038/s41598-021-85356-6

**Published:** 2021-03-15

**Authors:** Vajihe Azimian-Zavareh, Zeinab Dehghani-Ghobadi, Marzieh Ebrahimi, Kian Mirzazadeh, Irina Nazarenko, Ghamartaj Hossein

**Affiliations:** 1 Department of Animal Biology, School of Biology, University College of Science, University of Tehran, Tehran, Iran; 2 Applied Physiology Research Center, Cardiovascular Research Institute, Isfahan University of Medical Sciences, Isfahan, Iran; 3 Department of Stem Cells and Developmental Biology, Cell Science Research Center, Royan Institute for Stem Cell Biology and Technology, ACECR, Tehran, Iran; 4 Institute for Infection Prevention and Hospital Epidemiology, Medical Center - University of Freiburg, Faculty of Medicine, University of Freiburg, 79106 Freiburg, Germany

**Keywords:** Integrins, Morphogen signalling

## Abstract

Wnt5A signals through various receptors that confer versatile biological functions. Here, we used Wnt5A overexpressing human ovarian SKOV-3 and OVCAR-3 stable clones for assessing integrin expression, cell proliferation, migration, invasion, and the ability of multicellular aggregates (MCAs) formation. We found here, that Wnt5A regulates differently the expression of its receptors in the stable Wnt5A overexpressing clones. The expression levels of Frizzled (FZD)-2 and -5, were increased in different clones. However ROR-1, -2 expression levels were differently regulated in clones. Wnt5A overexpressing clones showed increased cell proliferation, migration, and clonogenicity. Moreover, Wnt5A overexpressing SKOV-3 clone showed increased MCAs formation ability. Cell invasion had been increased in OVCAR-3-derived clones, while this was decreased in SKOV-3-derived clone. Importantly, αv integrin expression levels were increased in all assessed clones, accompanied by increased cell attachment to fibronectin and focal adhesion kinase activity. Moreover, the treatment of clones with Box5 as a Wnt5A/FZD5 antagonist abrogates ITGAV increase, cell proliferation, migration, and their attachment to fibronectin. Accordingly, we observed significantly higher expression levels of ITGAV and ITGB3 in human high-grade serous ovarian cancer specimens and ITGAV correlated positively with Wnt5A in metastatic serous type ovarian cancer. In summary, we hypothesize here, that Wnt5A/FZD-5 signaling modulate αv integrin expression levels that could be associated with ovarian cancer cell proliferation, migration, and fibronectin attachment.

## Introduction

Ovarian cancer (OVC) is one of the most lethal gynecological malignancies that is frequently asymptomatic at early stages and low survival rate is mostly due by the development of a progressing metastatic disease^1,2^. The complexity of the OVC is further enhanced by tumor heterogeneity; particularly the coexistence of different cell populations within one single lesion gives rise to intra-tumoral heterogeneity (ITH). ITH has a crucial role in metastasis, invasion, tumor expansion, recurrence, and therapeutic resistance^3^. OVC metastasis is a multi-step process involving primary tumor cell’s shedding, resistance to anoikis, the formation of multicellular aggregates (MCAs) or spheroids, adhesion, disaggregation, and invasion of MCAs into omentum^4^. MCAs prepare an evolutionary privilege in tumor progression, as they are chemoresistant^5^, and are protected against anti-tumoral immune effector cells^6,7^. In addition, they exhibit high adhesion capacity to extracellular matrix (ECM) components of mesothelium through integrins^8,9^. Integrins are important mediators of signaling crosstalk between OVC cells and the mesothelium, by promoting MCAs formation^10^, dissemination, invasion, peritoneal metastasis^11,12^ and resistance of MCAs to anoikis^13^. The integrin α5β1 and αvβ6 has been reported as prognostic markers in a large cohort of OVC patients^10,14,15^.

Wnt5A belongs to the non-canonical Wnt pathway and mediates normal developmental processes, including self-renewal, proliferation, differentiation, migration, adhesion, cell polarity, and cytoskeletal reorganization^16^. Wnt5A exhibited dual function in tumors both tumor promoting and supressor^16^ by the different mechanism including the activity of its isoforms Wnt5A-long (Wnt5A-L) and Wnt5A-short (Wnt5A-S), binding to specific receptors, downstream effectors, exogenous inhibitors, and tumor microenvironments, as well as the extracellular matrix, particularly cell/tissue-tropic contexts^17^. We have previously reported that Wnt5A exhibits a tumor-promoting effect in ovarian cancer^18–20^. Several studies demonstrated the importance of Wnt5A on cell-to-substrate attachment in various cells and models ^21–26^, though; its exact molecular mechanism is still not understood.

Here, we examined the association between Wnt5A and integrin expression and/or activation using Wnt5A overexpressing SKOV-3 and OVCAR-3 clones. Furthermore, we assessed the Wnt5A effect on cell proliferation, MCAs formation ability, migration, and invasion by blocking Wnt5A/FZD-5 interaction with the small molecule Box5 or Wnt5A knock-down. Furthermore, different human serous histological subtypes were used to assess the expression of integrins compared to the normal ovary. Finally, an interaction between integrins and Wnt5A was evaluated by performing bioinformatics analysis.

## Results

### Different expression levels of Wnt5A interacting receptors in Wnt5A overexpressing clones

**Figure 1:**
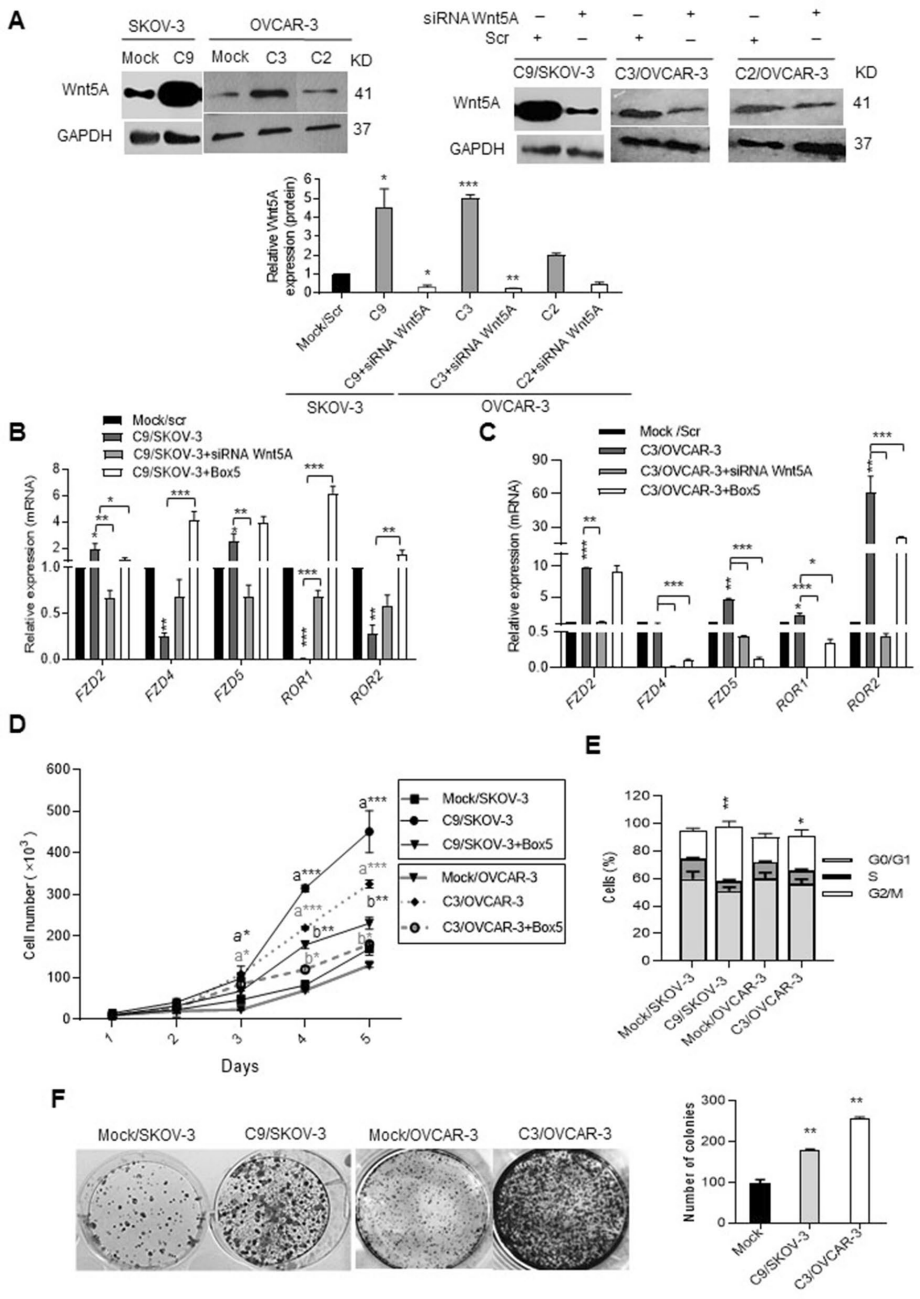
Wnt5A regulates its receptors and affects proliferation and clonogenicity of Wnt5A overexpressing clones. (**A**) Expression of Wnt5A in Clone 9 of SKOV-3 cells and Clone 3 and 2 of OVCAR-3 cells referred further as C9/SKOV-3 clone, C3/OVCAR-3 clone, and C2/OVCAR-3 clone was assessed by western blot (left and lower panel) compared to mock. The bands corresponding to GAPDH of the same blot was added separately. C9/SKOV-3, C3/OVCAR-3, and C2/OVCAR-3 clones were transfected with siRNA against Wnt5A showed a reduction of Wnt5A at protein levels (right and lower panels) compared to scramble (scr). (**B,C**) Wnt5A regulates its receptors as revealed by RT-qPCR analysis of frizzled-2 (FZD-2), frizzled-4 (FZD-4), frizzled-5 (FZD-5), ROR-1 and ROR-2 expression levels in C9/SKOV-3 and C3/OVCAR-3 clones in Wnt5A knock-down or Box5 treated clones relative to mock or scrambled (scr). GAPDH was used as an internal control. (**D**) Trypan blue exclusion test showed increased cell proliferation of C9/SKOV-3, and C3/OVCAR-3 clones compared to mock or Box5-treated cells. (**E**) The proportion of C9/SKOV-3, and C3/OVCAR-3 clones in the G2/M phase was increased compared to mock as revealed by cell cycle analysis. (**F**) Increased clonogenicity of C9/SKOV-3, and C3/OVCAR-3 clones compared to mock (left panel, scale bar: 100 µm). The right panel represents a quantitative assessment of colonies in the C9/SKOV-3 clone, C3/OVCAR-3 clone, and mock cells after 11 days. Results of RT-qPCR were normalized related to GAPDH used as an internal control. Mean ± SD from at least three independent experiments. a: compared to mock, b: compared to Wnt5A overexpressing clones *P < 0.05; **P < 0.01; ***P < 0.001 compared to mock.

Wnt5A overexpressing cells were subcloned and multiple clones showed high expression levels of Wnt5A (Supplementary Fig. S1A, S1B). Among the isolated clones, clone 9 in SKOV-3 cells named as C9/SKOV-3 clone and clone 3 in OVCAR-3 named as C3/OVCAR-3 clone with 4.5-fold increased Wnt5A expression levels (Fig. 1A, left, right and lower panel) and C2/OVCAR-3 clone cells with twofold increased levels of Wnt5A (Fig. 1, right and lower panels) were selected for further experiments. It is well known that the biological effect of Wnt5A is receptor-dependent, thus at first, we assessed Wnt5A’s receptor levels in these clones. We found a significantly increased level of FZD-5 by 2.7-fold in C9/SKOV-3 clone, by 4.0-fold in C3/OVCAR-3 clone, and by 8.2-fold in C2/OVCAR-3 clone. Similarly, FZD-2 expression levels were increased by 2.0-fold in C9/SKOV-3 clone, by 9.8-fold in C3/OVCAR-3 clone, and by 1.7-fold in C2/OVCAR-3 clone (Fig. 1B,C and Supplementary Fig. S1C). However, the expression levels of FZD-4 were decreased by 75%, 50%, and 40% in C9/SKOV-3, C3/OVCAR-3, and C2/OVCAR-3 clones, respectively, compared to mock. (Fig. 1B,C and Supplementary Fig. S1C). The expression profile of RORs receptors was different in each clone, showing significantly and strongly decreased levels of both ROR1 and ROR2 in the C9/SKOV-3 clone. Whereas, there were 63-fold and 3.0-fold increased levels of ROR2 in C3/OVCAR-3 and C2/OVCAR-3 clones, respectively compared to mock (*P* < 0.001) (Fig. 1B,C and Supplementary Fig. S1C). The expression levels of ROR1 were increased by 2.0-fold in the C3/OVCAR-3 clone (Fig. 1C) and strongly decreased by 90% in the C2/OVCAR-3 clone (Fig. S1C). These changes were reverted in Wnt5A knocked-down or Box5-treated clones (Fig. 1B,C, and Supplementary Fig. S1C).

There has been an increased cell proliferation (Fig. 1D) accompanied by increased mRNA levels of cell cycle markers c-myc, CCND1, and BIRC5 (Supplementary Fig. S1D,E), which reverted in the presence of Box5 or Wnt5A knocked-down cells (Fig. 1D and Supplementary Fig. S1D,E). Cell cycle analysis after 24 h showed a 2.0-fold and 1.4-fold increased cell proportion in the G2/M phase (*P* < 0.05) of C9/SKOV-3, and C3/OVCAR-3 clones, respectively, compared to mock (Fig. 1E). Moreover, when C9/SKOV-3 and C3/OVCAR-3 clones were cultured as single cells they showed a 1.9-fold and 2.7-fold increased ability to form colonies, respectively compared to mock (*P* < 0.01) (Fig. 1F). Similarly, there was a 1.9-fold increased number of colonies of C2/OVCAR-3 clone compared to mock (Data not shown).

### Wnt5A alters E-cadherin expression and morphology of Wnt5A overexpressing SKOV-3 clones

**Figure 2:**
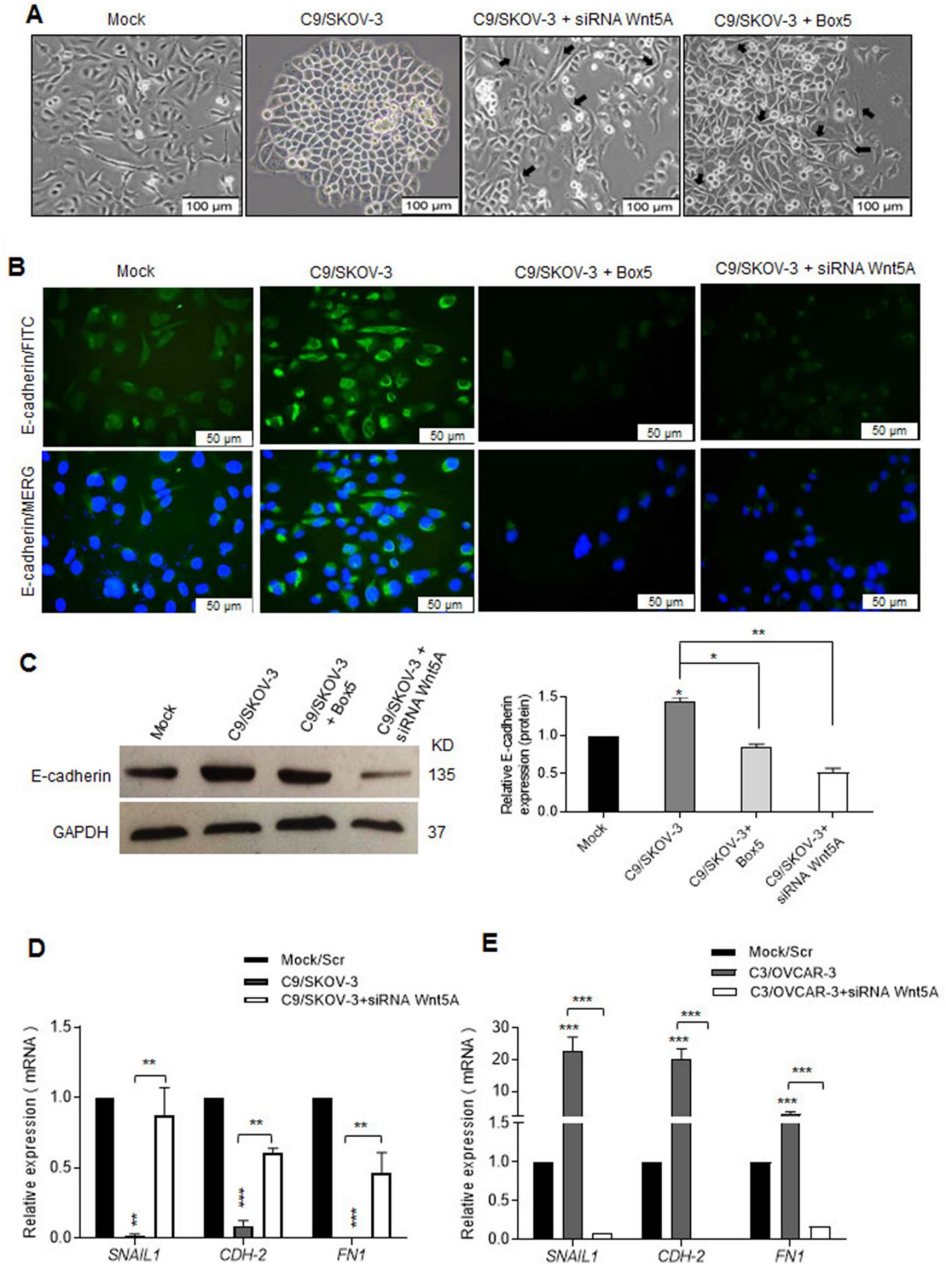
Wnt5A overexpression modulates mesenchymal markers and reverts the mesenchymal morphology of SKOV-3 cells. (**A**) C9/SKOV-3 clone showed epithelial-like morphology compared to spindle morphology of non-transfected cells. Wnt5A knock-down or blocking with Box5 partially rescued morphology alteration of C9/SKOV-3 clone (arrows) (scale bar: 100 µm). (**B,C**) C9/SKOV-3 clone showed increased E-cadherin immunostaining and expression compared to mock that was reverted using siRNA Wnt5A or Box5 (scale bar: 50 µm). The bands corresponding to GAPDH of the same blot was added separately. (**D**) Decreased mRNA levels of the mesenchymal markers CDH-2, SNAIL, and FN1 in C9/SKOV-3 clone compared to the mock which was reversed with siRNA Wnt5A. (**E**) The increased expression level of mesenchymal markers in C3/OVCAR-3 clone compared to mock which was reverted in Wnt5A knock-down. Results of RT-qPCR were normalized related to GAPDH used as an internal control. Mean ± SD of at least three independent experiments. *P < 0.05; **P < 0.01; ***P < 0.001 compared to mock.

Interestingly, the spindle-like morphology of SKOV-3 cells becomes epithelial-like in the C9/SKOV3 clone and other clones C1 and C4 (Fig. 2A and Supplementary Fig. S2A) with subsequent increased levels of E-cadherin (Fig. 2B,C). These changes were partially reverted in Wnt5A knocked-down or Box5-treated cells (Fig. 2A). Accordingly, Wnt5A overexpressing SKOV-3 clone showed a significant reduction of SNAIl1 (Snail1), CDH-2 (N-cadherin), and FN1 (fibronectin) as mesenchymal markers which were reverted in Wnt5A knocked-down cells (Fig. 2D and Supplementary Fig. S2B). While significantly increased expression levels of mesenchymal markers had been observed in Wnt5A overexpressing OVCAR-3 clones (Fig. 2E and Supplementary Fig. S2C) with no apparent morphological change (Supplementary Fig. S2D).

### Increased cell migration and alteration of cell invasion in Wnt5A overexpressing clones

**Figure 3:**
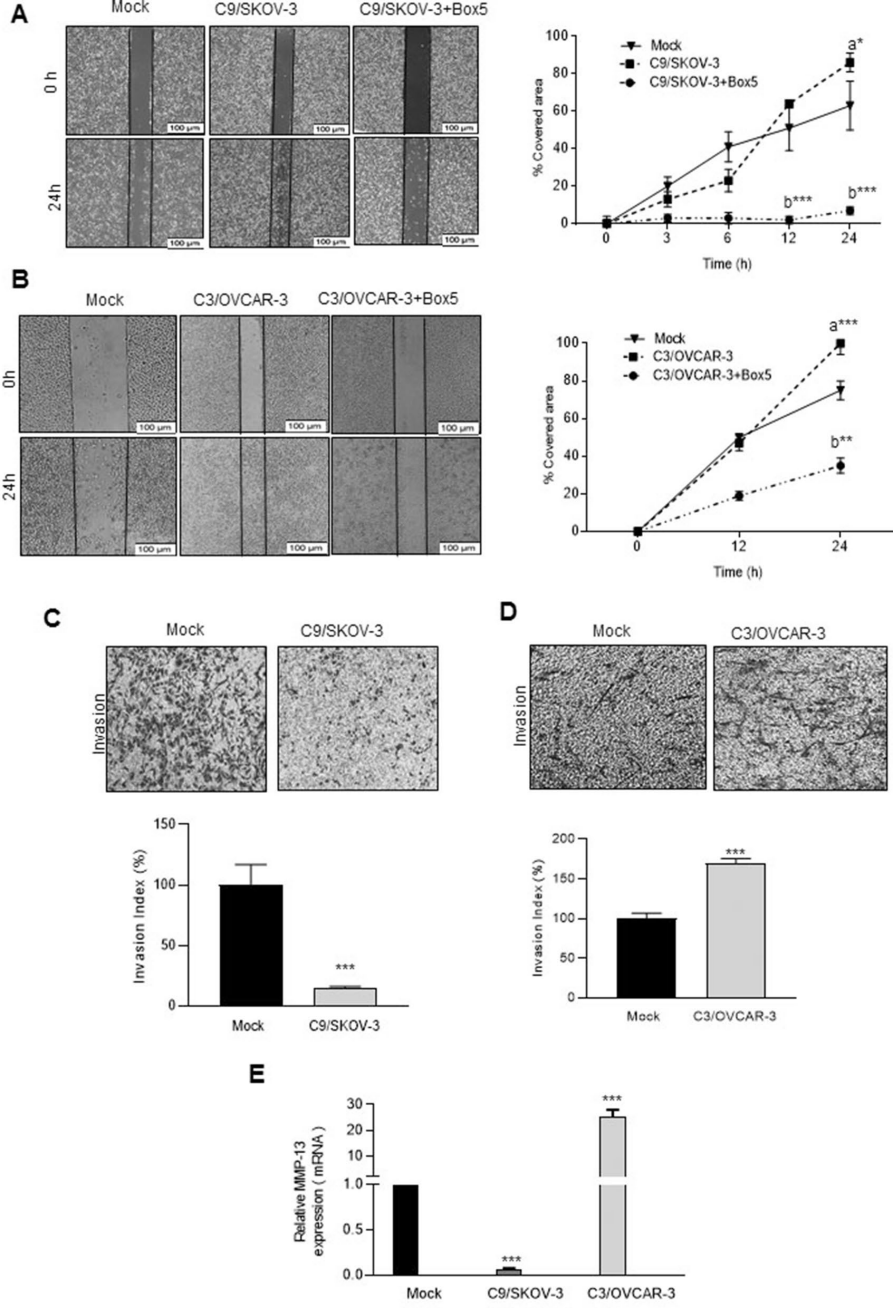
Wnt5A alters motility and invasiveness of Wnt5A overexpressing SKOV-3 and OVCAR-3 clones. (**A**) Increased migration of C9/SKOV-3 clone which was abrogated in the presence of Box5 (left panel, scale bar: 100 µm). The right panel shows the migration rate of cells that were assessed based on the distance of the selected wounded area at time intervals of 0, 3, 6, 12, and 24 h, and the percent of wound closure was determined for each time point. (n = 3, mean ± SD). (**B**) Wound-healing analysis of C3/OVCAR-3 clone during a 24-h time course in the presence or absence of Box5. Increased motility of C3/OVCAR-3 clone was abrogated in the presence of Box5 (250 µM) (left and right panel, scale bar: 100 µm). (**C**) C9/SKOV-3 clone showed decreased cell invasion compared to mock (upper panel, photos are representative of one of three performed experiments). The lower panel shows quantification of cell invasion by counting cells at ten random fields (n = 3, mean ± SD; scale bar: 100 µm). (**D**) C3/OVCAR-3 clone showed increased cell invasion compared to mock (upper panel, photos are representative of one of three performed experiments). The lower panel shows quantification of cell invasion by counting cells at ten random fields (n = 3, mean ± SD; scale bar: 100 µm). (**E**) Decreased and increased mRNA levels of MMP-13 in C9/SKOV-3, and C3/OVCAR-3 clones, respectively compared to mock. Results of RT-qPCR were normalized related to GAPDH used as an internal control. Mean ± SD of three independent experiments. a: compared to mock, and b: compared to Wnt5A overexpressing clones. *P < 0.05; **P < 0.01; ***P < 0.001 compared to mock.

There was increased cell migration of C9/SKOV-3, and C3/OVCAR-3 clones compared to mock (Fig. 3A,B). This was decreased by 79% and 65% in Box5-treated C9/SKOV-3, and C3/OVCAR-3 clones respectively, compared to untreated cells (*P* < 0.01) (Fig. 3A,B left and right panels). Also, the treatment of mock cells with conditioned medium (C.M) derived from C9/SKOV-3 or C3/OVCAR-3 clones led to increased cell migration which was abrogated in the presence of Box5 (Supplementary Fig. S3A,B, left and right panels). Surprisingly, cell invasion of the C9/SKOV-3 clone was decreased by 80% (*P* < 0.001) compared to mock (Fig. 3C). However, there were1.7-fold (*P* < 0.001) and, 1.2-fold (*P* < 0.05) increased cell invasion in the C3/OVCAR-3, and C2/OVCAR-3 clones compared to mock (Fig. 3D and Supplementary Fig. S4A). As shown in Fig. 1B,C, ROR1 and ROR2 expression levels were down-regulated in Wnt5A overexpressing C9/SKOV-3 clone but not in Wnt5A overexpressing OVCAR-3 clones this has prompted us to assess the levels of MMP-13 as a downstream target of Wnt5A/ROR2 signaling which played a role in cell invasion ^27,28^. A noticeable decreased MMP-13 expression level was found in the C9/SKOV-3 clone compared to mock while; this was increased in the C3/OVCAR-3 clone relative to mock (Fig. 3E).

### Wnt5A alters SKOV-3 MCAs formation ability and compactness

**Figure 4:**
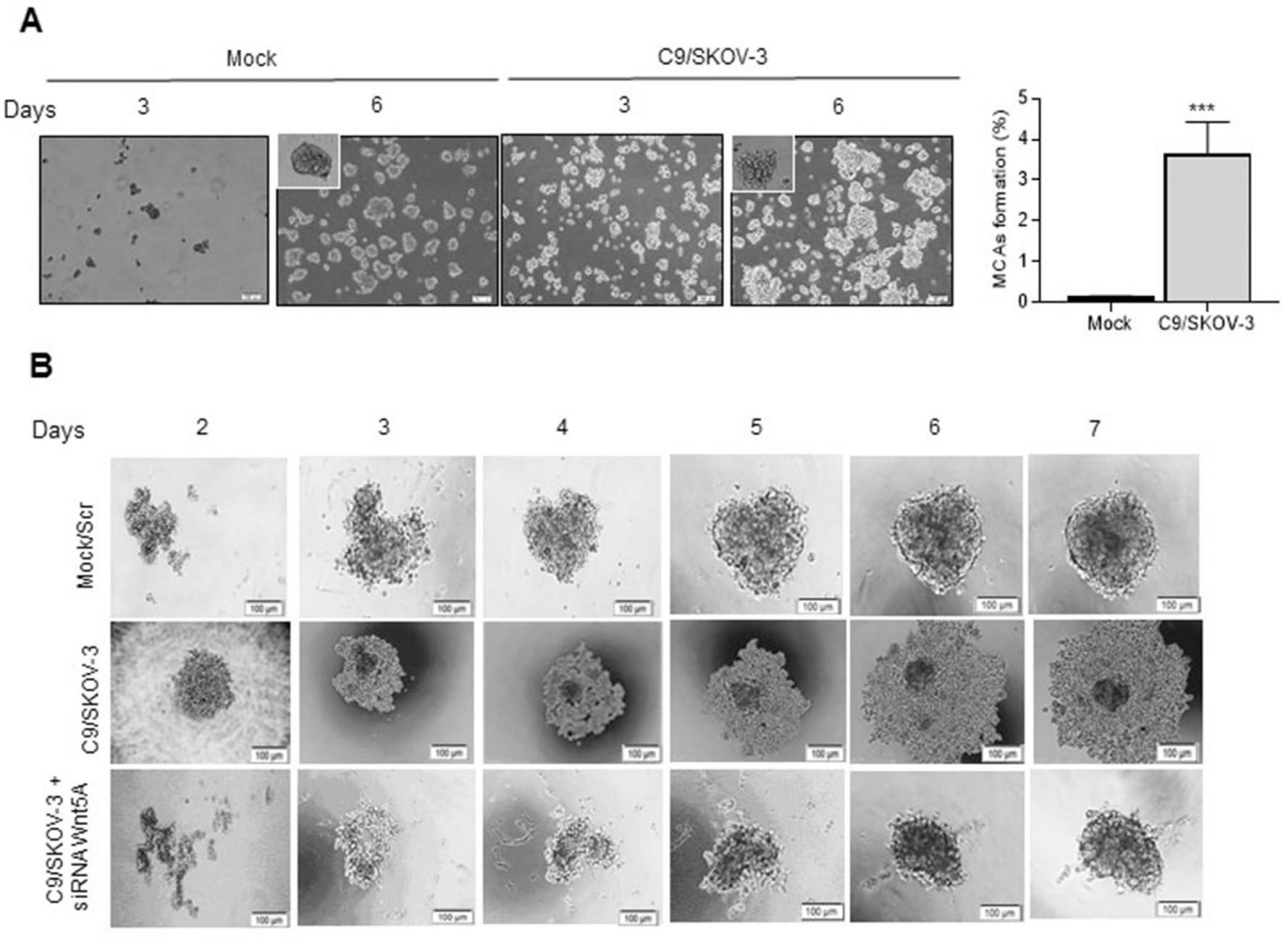
Wnt5A increases MCAs formation and decreases the compactness of C9/SKOV-3 MCAs. (**A**) MCAs morphology during 3 and 6 days culture (left panel). MCAs formation rate of C9/SKOV-3 clone vs. mock cells (right panel) was quantitated after 6 days in 50 µl droplet under an inverted microscope. (**B**) C9/SKOV-3 clone was transfected with siRNA against Wnt5A or scramble siRNA (25 nM) and the formation of C9/SKOV-3 MCAs was followed over 7 days. Wnt5A knockdown reverted the loose structure of MCAs formed by the C9/SKOV-3 clone. Photos represent one of the three independently performed experiments (scale bare: 100 µm). *P < 0.05; **P < 0.01; ***P < 0.001 compared to mock.

The formation of MCAs is one of the hallmarks of a metastasizing ovarian carcinoma^29^, which supports the survival of the tumor cells in ascites and protects them from chemotherapy^30–32^. Here we found that Wnt5A overexpression led to a significant increase by 3.6-fold in MCAs formation ability of C9/SKOV-3 clone compared to mock (*P* < 0.001, Fig. 4A, left and right panels); however, those MCAs were looser than mock MCAs (Fig. 4A) which was abrogated in the Wnt5A knocked-down C9/SKOV-3 clone (Fig. 4B, lower panel). Moreover, we found an increased level of Wnt5A, c-myc, and CDH-1 expression from day 3 to day 6 in C9/SKOV-3 MCAs compared to mock MCAs (Supplementary Fig. S4B).

### Wnt5A modulates integrin expression and increase cell attachment to fibronectin and laminin

**Figure 5:**
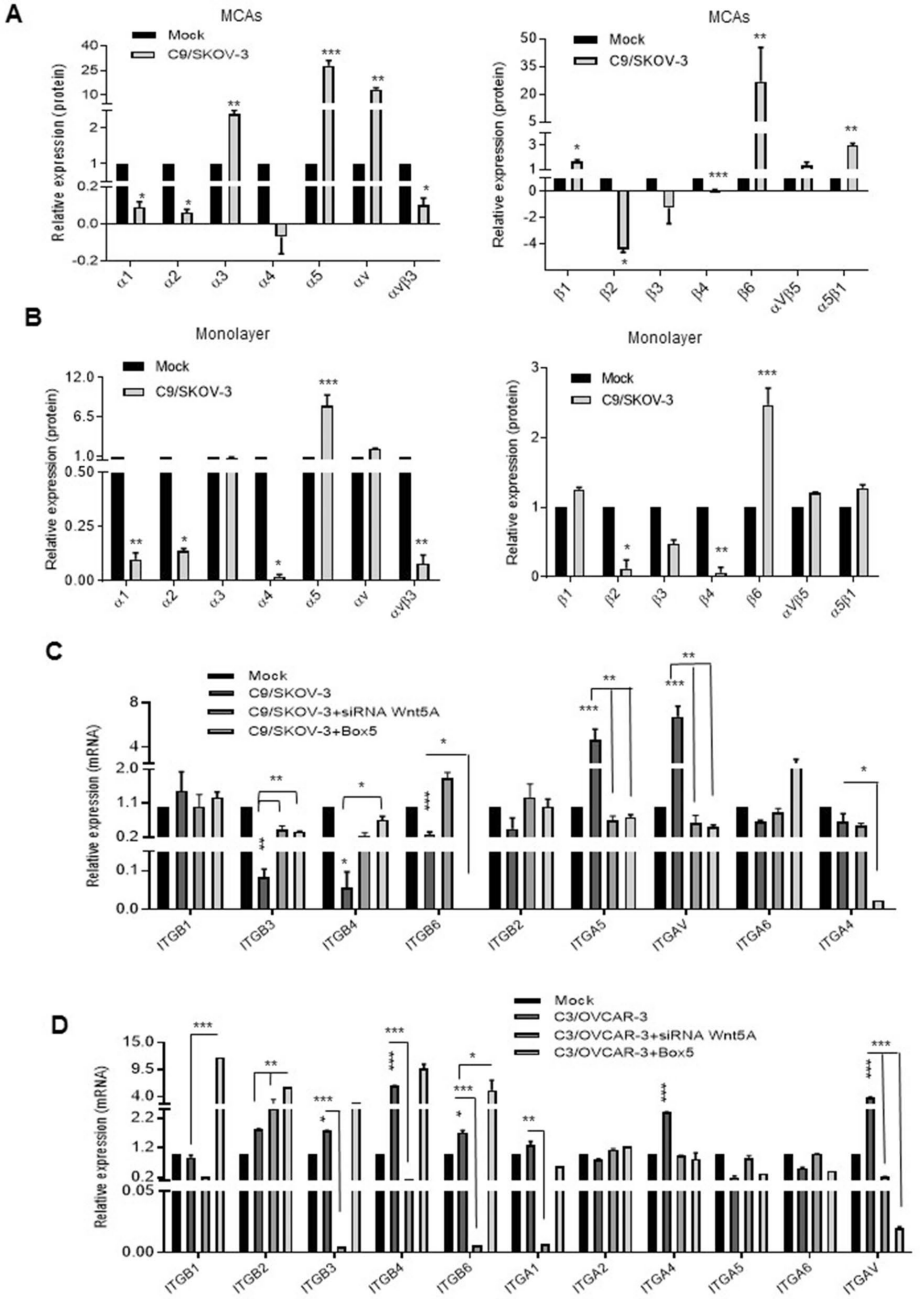
Wnt5A modulates integrin expression in Wnt5A overexpressing clones in both 2D and 3D cell culture. (**A**) The integrin expression level was assessed in 3D and (**B**) 2D Wnt5A overexpressing C9/SKOV-3 clone and mock cells by using the Alpha/Beta Integrin-Mediated Cell Adhesion Array Combo Kit. Values were normalized related to mock from two independent experiments each performed in duplicate (mean + SD). (**C**) RT-qPCR analysis of integrin subunits in C9/SKOV-3 clone with or without siRNA Wnt5A or Box5 relative to mock or scrambled (scr). (**D**) RT-qPCR analysis of integrin subunits in C3/OVCAR-3 clone with or without siRNA Wnt5A or Box5 relative to mock or scrambled (scr). GAPDH was used as an internal control and the data represent mean ± SD (n = 3) *P < .05; **P < .01; ***P < .001 compared to mock.

We have previously demonstrated an increased substrate-dependent adhesion in Wnt5A overexpressed SKOV-3 cells in 2D culture^20^. This may raise the question of whether Wnt5A could modulate integrin expression and/or activation. Here, we performed the integrin array assay and immunofluorescence analysis of phospho-focal adhesion kinase (pTyr^397^-FAK). We found a greater modulation of integrin expression in C9/SKOV-3 MCAs compared to monolayer (Fig. 5A vs. 5B). The expression levels of the following integrins had been increased in Wnt5A overexpressing C9/SKOV-3 MCAs: α3 (2.4-fold, *P* < 0.001), α5 (28-fold, *P* < 0.001), αv (13.5-fold, *P* < 0.001), β1 (1.6-fold, *P* < 0.01), β6 (27-fold, *P* < 0.01) and α5β1 (3.0-fold, *P* < 0.001) compared to mock MCAs (Fig. 5A, right and left panels). In comparison, C9/SKOV-3 monolayer showed increased levels of α5 (8.1-fold, *P* < 0.001), αv (2.1-fold, *P* < 0.001) and β6 (2.5-fold, *P* < 0.001) integrins compared to mock (Fig. 5B, right and left panels). Most of the changes in integrin expression were reverted in the Wnt5A knocked-down C9/SKOV-3 clone or in the presence of Box5 (Fig. 5C).

There was a greater modulation of integrin expression in the C3/OVCAR3 clone compared to C2/OVCAR-3 clone (Fig. 5D vs. Supplementary Fig. S5A). In C3/OVCAR-3 clone there were significantly increased expression levels of ITGA1, ITGA4, ITGAV, ITGB2, ITGB3, ITGB4, and ITGB6 (Fig. 5D). These changes were reverted in Wnt5A knocked-down C3/OVCAR-3 clone however, most integrin expression remains high in the presence of Box5 excepting ITGAV compared to mock (Fig. 5D). It is worth noting that ITGAV expression levels have been significantly increased in both Wnt5A overexpressing SKOV-3 and OVCAR-3 clones, which were reversed both in Wnt5A knocked-down or Box5-treated clones (Fig. 5 and Supplementary Fig. S5A).

**Figure 6:**
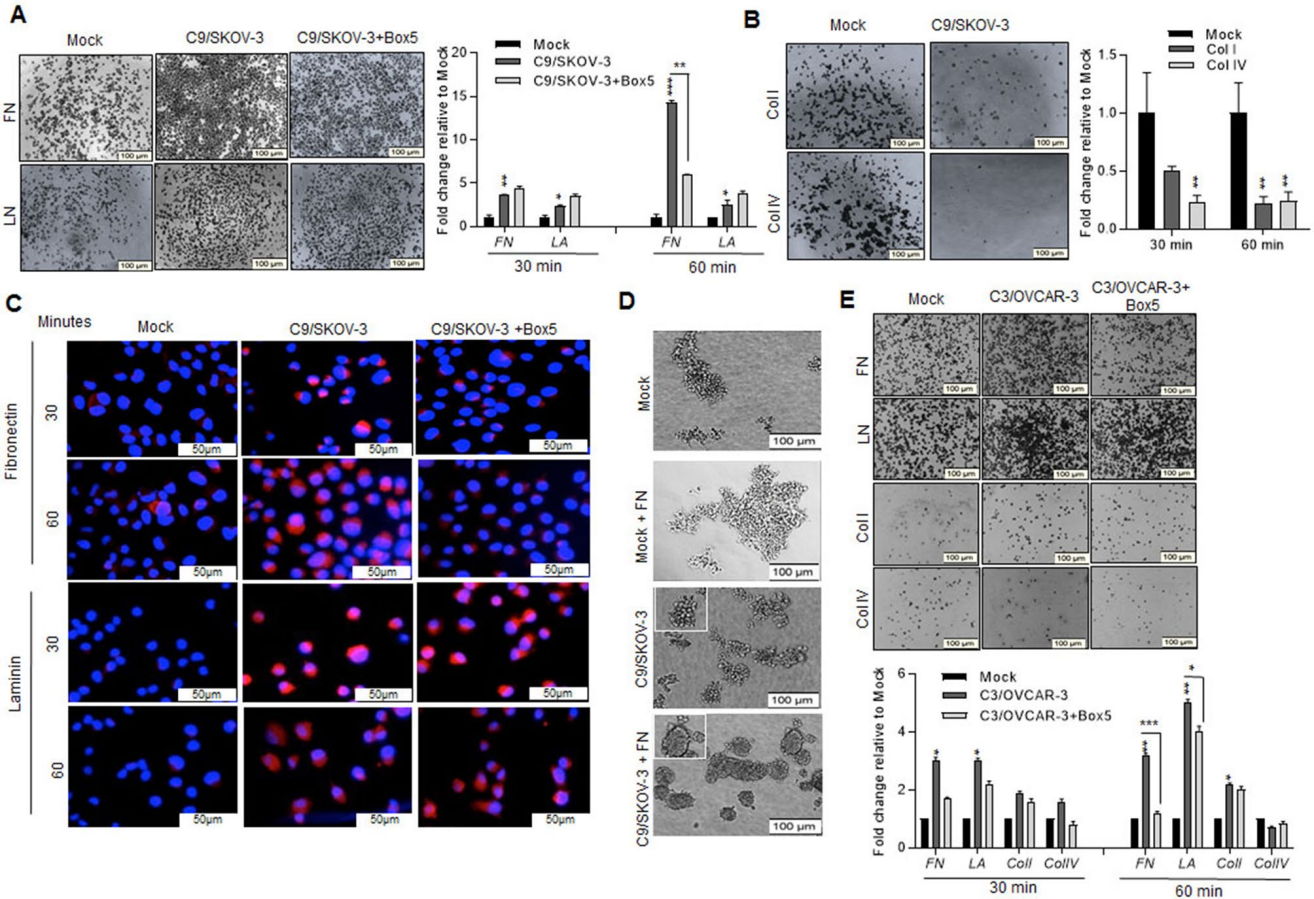
fibronectin- and laminin-dependent adhesion of Wnt5A overexpressing clones and subsequent FAK activation. (**A**) C9/SKOV-3 clone or mock cells were seeded on fibronectin (FN)—or laminin (LN)-coated wells in the presence or absence of Box5 and the percent of adhered cells was assessed after 30 or 60 min. Left panel: Photos represent one of the three independent experiments after 60 min (scale bar: 100 µm). Right panel: Adhered cells were stained with crystal violet and quantitated. (**B**) C9/SKOV-3 clone or mock cells were seeded on collagen type-I or IV-coated wells and the percent of adhered cells was assessed after 30 or 60 min. Left panel: Photos represent one of the three independent experiments (scale bar: 100 µm). Right panel: Adhered cells were stained with crystal violet and quantitated. (**C**) Mock or C9/SKOV-3 clone with or without Box5 was seeded on FN- or LN-coated wells and immunostained with anti- pTyr^397^-FAK after 30 or 60 min. Photos are representative of three independent experiments (scale bar: 50 µm). (**D**) C9/SKOV-3 clone and Mock cells were mixed with 25 µg/ml of fibronectin (FN) and were cultured on agarose-coated 12 well plates for 24 h. Increased compaction of cells was shown in the presence of FN in Wnt5A overexpressed cells compared to mock. (**E**) C3/OVCAR-3 clone or mock cells were seeded on FN, LN-, Collagen- (I and IV types) coated wells in the presence or absence of Box5, and the percent of adhered cells was assessed after 30 or 60 min. Upper panel: Photos represent one of the three independent experiments after 60 min (scale bar: 100 µm). Lower panel: Adhered cells were stained with crystal violet and quantitated. The results are expressed as mean + SD relative to mock from three independent experiments each performed in triplicate. (Scale bar: 100 µm). *P < .05; **P < .01; ***P < .001 relative to mock.

Next, we assessed the cell adhesion of Wnt5A overexpressing clones to extracellular matrix components which showed increased cell adhesion to fibronectin (FN) and laminin (LN) compared to mock cells (Fig. 6A, upper and lower panels). Meanwhile, Box5 reversed adhesion to FN (Fig. 6A lower panel), and C9/SKOV-3 clone cell adhesion to Collagen type –I (Col. I) and –IV (Col. IV) was decreased up to 80% (1 h incubation) compared to mock (Fig. 6B). Subsequently, C9/SKOV-3 clone cell adhesion to FN or LN showed increased pTyr^397^-FAK immunostaining (Fig. 6C), indicating activation of FAK, as an important regulator of integrin signaling^33^. Moreover, FN-dependent FAK activation had been reduced in the presence of Box5 (Fig. 6C). In line with our data here, loose C9/SKOV-3 MCAs became more compact upon the addition of FN into the C9/SKOV-3 clone (Fig. 6D). Similarly, C3/OVCAR-3 clone cell adhesion to FN and LN has been significantly increased and its adhesion to FN was abrogated in the presence of Box5 (Fig. 6E). There was an increased C3/OVCAR-3 clone cell adhesion to Col I and Col IV that was not modulated by the presence of Box5 (Fig. 6E). Altogether, our results showed increased adhesion of Wnt5A overexpressing clones to FN that may require Wnt5A/FZD-5 signaling as Box5 reverts it.Wnt5A alters E-cadherin expression and morphology of Wnt5A overexpressing SKOV-3 clones.

### Wnt5A correlates positively with ITGA5, ITGAV, ITGA4, and ITGB6 expression in the serous type ovarian cancer

**Figure 7:**
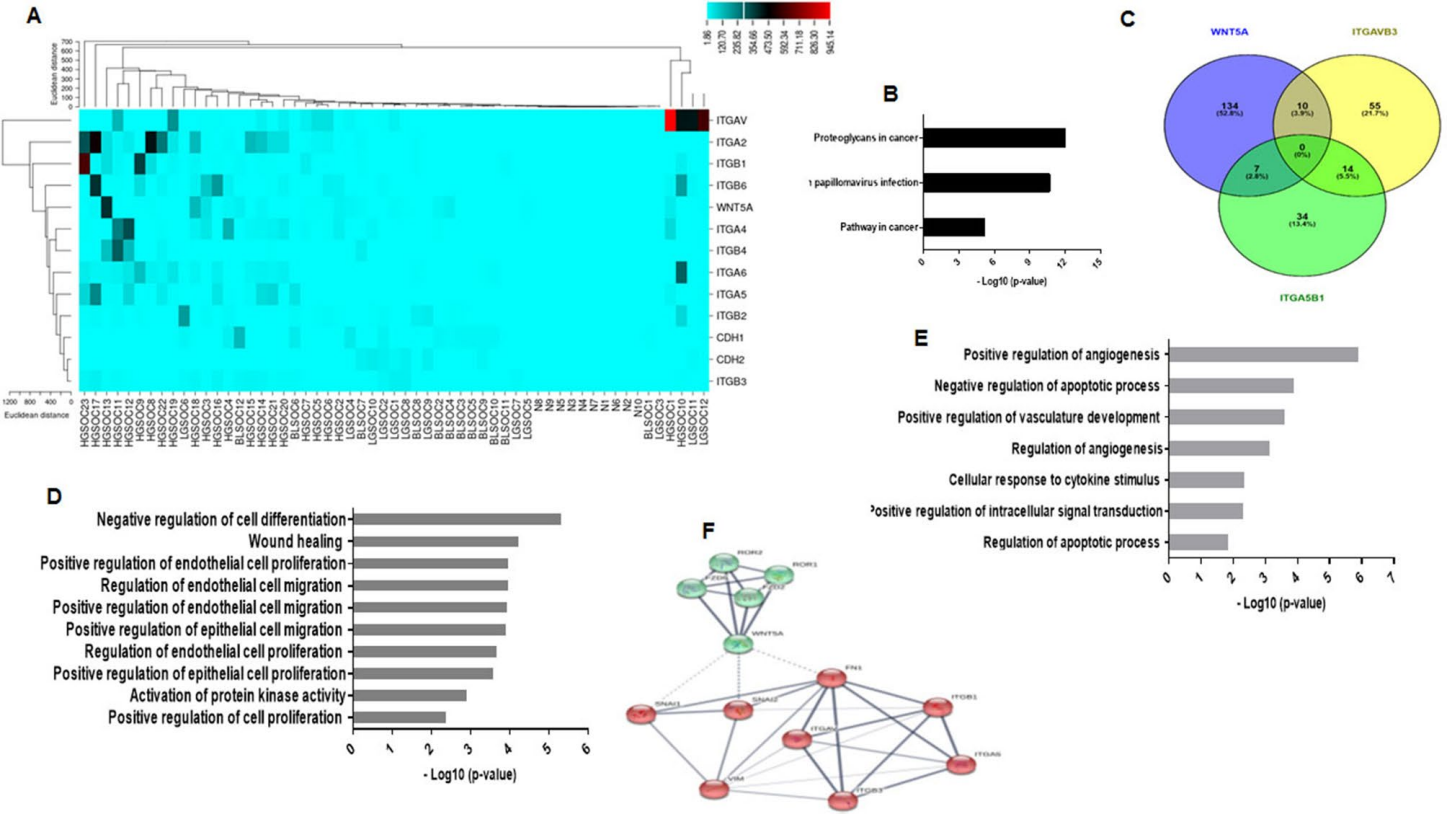
Venn diagram of common GO (BP) and Protein–protein interaction network for Wnt5A, and integrins and their expression levels in human serous histological subtypes. (**A**) The heatmap shows the expression levels of integrins, Wnt5A, and cadherins obtained by hierarchical cluster analysis. Each column in the figure represents a sample, and each row represents a gene. The colors in the graph indicate the magnitude of gene expression in the sample. The black-red gradient indicates that the genes are medium–high expressed in the samples, and the blue indicates that the gene expression is low. (**B**) Common KEGG pathways analysis of ITGAV, ITGA5, and Wnt5A (**C**) All GO (BP) are grouped into three comparison groups represented by three circles. The overlapping portions of the different circles represent the number of GO (BP) common to these comparison groups. (**D**) Common GO (BP) for Wnt5A and ITGAVB3. (**E**) Common GO (BP) for Wnt5A and ITGA5B1. (**F**) Protein–protein interaction (PPI) network of Wnt5A and ITGAV, ITGB3, ITGA5, and ITGB1, the thickness of the edge indicates a strong interaction between the two proteins. N: normal; BL: Borderline; LGSOC: Low grade serous ovarian cancer; HGSOC: High grade serous ovarian cancer.

We drew the heatmap plot to classify the up-regulated and down-regulated analyzed genes including integrins, CDH-1, CDH-2, and Wnt5A in different subtypes of serous ovarian cancer and normal ovary (Fig. 7A). Next, a two factorial experimental design was developed to evaluate the difference in gene expression between groups which showed that the assessed genes were significantly different between normal and cancerous groups (*P* < 2.2 × 10–16). CDH-1 and CDH-2 were significantly lower in the HGSOC group compared to other groups (Fig. 7A). In HGSOC, we found a higher expression levels of ITGA2 (*P* < 0.001), ITGA4 (*P* < 0.05), ITGA5 (*P* < 0.05), ITGB6 (*P* < 0.01) compared to other groups (Fig. S6A-D). In HGSOC, ITGAV expression levels were higher (*P* < 0.05, compared to BL and normal groups), and ITGB1 and ITGB3 were higher compared to normal and LGSOC groups (*P* < 0.01 and *P* < 0.05, respectively) (Fig. S6E-G). We further investigated the existence of a relationship between Wnt5A, and integrins in the metastatic groups (LGSOC + HGSOC). We found that Wnt5A was positively correlated with ITGA4 (r = 0.52, *P* = 0.02), ITGA5 (r = 0.39, *P* = 0.05), ITGAV (r = 0.44, *P* = 0.03) and ITGB6 (r = 0.53, *P* = 0.01). Our results with human specimens partially support our data from in vitro experiments and suggest that Wnt5A in ovarian cancer exerts a modulatory role on integrin expression.

### GO enrichment biological process and KEGG analysis reveals that αvβ3 integrin and Wnt5A contribute to epithelial cell migration and proliferation

We analyzed significantly differentially expressed (DE) integrins (ITGA2, ITGA5, ITGAV, ITGB1, ITGB2, ITGB3, and ITGB6) in HGSOC and Wnt5A for their contribution in GO terms biological process (BP) and KEGG pathways and the key GO terms and KEGG pathways were extracted. Our analysis revealed 3 enriched KEGG pathways shown in (Fig. 7B) which involve proteoglycans in cancer and pathways in cancer. Among DE integrins in HGSOC, we found that Wnt5A and ITGAV are involved in two enriched common BP as well as for ITGA5 and Wnt5A (http://amp.pharm.mssm.edu/Enrichr). Moreover, Wnt5A showed seven enriched common BP with ITGB1 and 9 enriched common BP with ITGB3 (http://amp.pharm.mssm.edu/Enrichr). Interestingly, ITGAV and Wnt5A are involved in the negative regulation of cell differentiation (GO: 0045596) and positive regulation of cell proliferation (GO: 0008284). It is well known that αvβ3 and α5β1 interact with FN^34^. Since in our model Wnt5A overexpressing clones showed increased adhesion to FN which could be reversed with Box5, this further encourages us to perform GO (BP) analysis for common BPs of αvβ3and Wnt5A (Fig. 7C,D) and 7 common BPs for α5β1 and Wnt5A (Fig. 7C,E). In line with our results here, our analysis revealed that both Wnt5A and αvβ3 significantly contribute to positive regulation of epithelial cell migration (GO: 0010594), and proliferation (GO: 0010634), negative regulation of cell differentiation (GO: 0045596), activation of protein kinase activity (GO: 0032147) (Fig. 7D). Further analysis of common BPs for α5β1 and Wnt5A showed their contribution to the regulation of angiogenesis (GO: 0045765), regulation of apoptotic process (GO: 0042981), positive regulation of intracellular signaling (GO: 1,902,533), and cellular response to cytokine stimulus (GO:0071345). (Fig. 7E). Moreover, Wnt5A showed significant interaction with ITGAV, ITGB3, ITGA5, and ITGB1 through FN, SNAI1, and SNAI2 (*P* < 1e−16) (Fig. 7F).

### Inhibition of αv integrin abrogates Wnt5A-induced cell proliferation and migration

**Figure 8:**
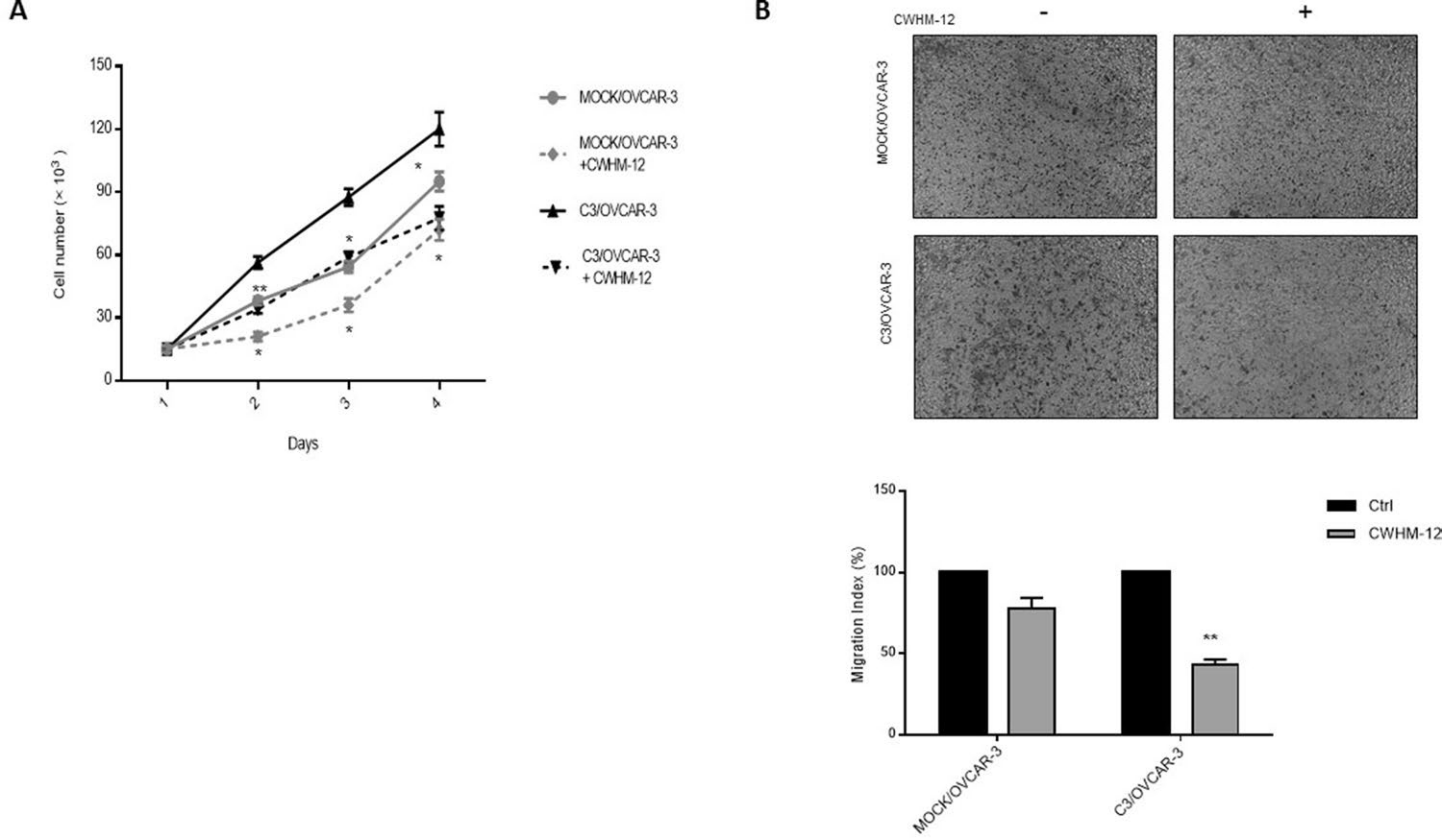
Inhibition of integrin αv decreases proliferation and migration of Wnt5A overexpressed clones. C3/OVCAR-3 clone or mock cells were treated with 25 μM CWHM- 12 as a specific inhibitor of αv integrin (**A**) Trypan blue exclusion (**B**) Transwell cell migration assay (scale bar: 100 μM). The results are expressed as mean + SD relative to untreated cells from three independent experiments each performed in triplicate. *P < .05; **P < .01.

Since we found that Wnt5A and ITGAV contribute to cell proliferation and migration (Fig. 7D), it was tempting to investigate the effect of specific αv inhibitor CWHM-12 on Wnt5A overexpressed cells. We found decreased cell proliferation in the presence of CWHM-12. (Fig. 8A). Moreover, cell migration was decreased by 22% and 57% in mock, and C3/OVCAR-3 clone in the presence of CWHM-12, respectively compared to untreated cells (Fig. 8B upper and lower panels). This supports the hypothesis that Wnt5A modulates cell proliferation and migration of ovarian cancer cells through up-regulation of ITGAV expression.

## Discussion

Analysis of a large cohort of OVC patients and evaluation of data obtained from The Cancer Genome Atlas (TCGA) found up-regulation of Wnt5A expression in all major histotypes relative to benign controls^16,35^. Furthermore, Wnt5A protein is present in ovarian tumor ascites^16^, supporting its contribution to the ovarian cancer progression^36^. Our previous report showed the role of Wnt5A in the regulation of substrate-dependent adhesion and migration^20^, though, the exact mechanism by which Wnt5A promotes cell adhesion remains unclear.

Since Wnt5A signals through different receptors related to its versatile biological effect, therefore, here we investigate first whether Wnt5A could regulate its receptors in our model. We found up-regulation of FZD-2 and FZD-5 and down-regulation of FZD-4 but ROR1 and ROR2 have been differently modulated in the Wnt5A overexpressing clones. Interestingly, changes in the expression levels of these receptors were rescued either by Wnt5A knock-down or inhibiting Wnt5A signaling by the Box5 antagonist which is expected to exert its effect via a direct FZD-5 binding^37,38^. Here, we report increased cell adhesion to FN, increased cell migration, and cell proliferation, which had been reversed in the presence of Box5. In line with our findings here, it has been reported that Wnt5A/FZD-5 signaling increased melanoma cell adhesion and migration, which was reversed in the presence of Box5^37,39^. Our findings here are further supported by the fact that Knockdown of either ROR1 or ROR2 alone did not affect cell adhesion to collagen or fibronectin, suggesting that the ROR receptors do not play a major role in regulating ovarian cancer cell adhesion^35^. However, either ROR1 or ROR2 contributes to OVC cell invasion^35^ corroborating with our observation here that C9/SKOV-3 clone with down-regulated RORs receptors showed reduced cell invasion but a higher invasive index in Wnt5A overexpressing OVCAR-3 clones with up-regulated ROR2 receptor was observed. Furthermore, our findings here are substantiated by a significantly decreased level of ROR-2-inducedn MMP-13 in C9/SKOV-3 clone. Of particular note, MMP-13 is reported to be associated with the invasion ability of osteosarcoma, and thyroid cell carcinoma^27,28^.

Moreover, a recent study showed that stable knockdown of ROR2 led to reverse the epithelial-mesenchymal transition in SKOV3 cells^40^. This may support our finding here that the decreased levels of ROR2 in the C9/SKOV-3 clone could be associated with its epithelial-like morphology accompanied by increased levels of E-cadherin. Wnt5A is associated with EMT in ovarian cancer^36,41^ however, here, we found more motility in C9/SKOV-3 clone with increased E-cadherin levels. This may propose that Wnt5A-mediated EMT may not necessarily be associated with the down-regulation of E-cadherin. This hypothesis may be further supported by the fact that other studies report a similar phenomenon in colorectal cancer^42^ in addition to, the observed mesenchymal phenotype in Wnt5A knocked-down breast cancer cells^43^. Of particular interest, we observed cytoplasmic E—cadherin immunostaining in C9/SKOV-3 clone, and in our 3D model, C9/SKOV-3 MCAs showed looser cell–cell contact and were more sensitive to Paclitaxel compared to mock (Data not shown) which may suggest that E-cadherin can be internalized and stabilized in these cells rather than forming a cell–cell junction.

To the best of our knowledge, there is no report about Wnt5A regulatory role on integrin expression and/or activation in ovarian cancer. Here we show for the first time that Wnt5A overexpression leads to the increased expression levels of αv and α5 integrins which could heterodimerize with β1, β3, β5, β6, and β8 integrins interacting with FN or LN and subsequent activation of FAK in a substrate-dependent manner. FAK is activated through autophosphorylation at Tyr^397^, which is initiated by the integrin engagement with its ligand, and the turnover of focal adhesions is required for a cell to spread and migrate^44^. One study showed that the expression of integrins ITGA1, ITGA2, and ITGAV increased over time and correlated with increased Wnt5A expression in human mesenchymal stem cell (HMSCs) differentiation to osteoblastic lineage and treatment of HMSCs with Wnt5A, increased integrin expression^25^. In this study, we found up-regulation of ITGAV in Wnt5A overexpressing clones and its reversal in the presence of Box5, suggesting that Wnt5A/FZD-5 signaling could mediate its increase. In line with this hypothesis, FZD-5 increased adhesion to FN and vitronectin in ovarian cancer cells^45^. We also found here increased expression levels of FZD-2 in Wnt5A overexpressing clones. It is worth noting that the dynamics of Wnt5A-dependent focal adhesion activity had been regulated through FZD-2, Dvl, and APC^21^. Future studies by targeting FZD-2 or FZD-5 may unravel and shed light on the understanding of Wnt5A's modulatory role in integrin expression.

Integrins mediate the initial aggregation of MCAs^31,46,47^. Interestingly, this study showed that the fold change of α5, αv, and α5β1 FN binding integrin subunits were remarkably higher in MCAs compared to monolayer C9/SKOV-3 clone. It has been reported that the interaction between α5β1 and FN is involved in the formation, adhesion, and disaggregation of ovarian cancer MCAs^31,32,48,49^. Accordingly, we showed here that Wnt5A overexpressing cells were able to form compact MCAs in the presence of FN. In line with these in vitro data, we found a positive relationship between Wnt5A and α5, αv, and β6 expression in the metastatic serous ovarian cancer groups. This is consistent with the emerging data suggesting that αvβ 6 and α5β1 integrins regulate invasion and metastases of ovarian cancer^15,50^.

Here, we found increased cell proliferation in the assessed Wnt5A overexpressing clones which showed increased FZD-2 and FZD-5 mRNA levels. One study has shown that FZD-2 stimulated cell proliferation and promoted cell migration in high-risk neuroblastoma by interfering with _β-catenin-dependent and β-catenin-independent signaling pathways^51^. Moreover, Wnt5A/FZD-2 signaling could activate Src family kinases (SFKs) and induces cervical, lung, and esophageal cancer cell proliferation^52^. It should be noted that SFKs primarily transmit signals downstream of receptor tyrosine kinases (RTKs) and integrins to regulate cell proliferation, motility, and survival^53^. Another important finding in the present study is that Wnt5A/FZD-5-mediated ITGAV up-regulation contributes to cell proliferation and migration. Furthermore, knockout of FZD-5 robustly inhibited cell growth in breast cancer cells and RNF43-mutant pancreatic ductal adenocarcinoma cells^54,55^. In line with these findings, we showed here, that antagonizing Wnt5A/FZD-5 with Box5 abrogates increased cell proliferation in Wnt5A overexpressing clones. Since GO (BP) enrichment analysis revealed positive regulation of cell proliferation by Wnt5A and ITGAV we could also suggest that Wnt5A-induced ITGAV contributes to cell proliferation in our model.

In conclusion, we identified that Wnt5A affects integrin expression, and particularly Wnt5A/FZD-5 signaling affects αv integrin expression, cell proliferation, migration, and invasion. In particular, Wnt5A-induced ITGAV may be important in increasing tumor cell adhesion, proliferation, and migration contributing to OVC progression.

## Methods

All methods were carried out following relevant guidelines and regulations and, all experimental protocols were approved by the University of Tehran, college of science and Royan institute. For human specimens, informed consent was obtained from all subjects.

### Cell line and culture conditions

SKOV-3 and OVCAR-3 cell lines (ovarian adenocarcinoma) were provided by Dr. Zarnani A.H from Avicenna research institute and cultured as previously described^20^. For 3D culture, cells were seeded in plates coated with 1% low melt agarose (IBI SCIENTIFIC, Tryon, NC, USA) in complete medium (RPMI containing 10% FBS, 1% L-glutamine, 1% penicillin/streptomycin).

### Stable overexpression of Wnt5A and transient Wnt5A gene knockdown

SKOV-3 and OVCAR-3 cells with 70–90% confluency were transfected with a pcDNA3.2/V5-DEST-Wnt5A plasmid (kindly provided by the laboratory of Dr. Marian L. Waterman, Institute for Immunology, UCI, CA, USA) or with an empty vector pcDNA3.2/V5-DEST (mock) using Lipofectamine 3000 and P3000 (Life Technologies, Inc., USA) according to the manufacturer’s protocol. After 48 h, selection reagent G418 (1 mg/ml, Sigma-Aldrich, Germany) was added to select the stably transfected clones for 17 consecutive days. The Wnt5A overexpressing SKOV-3 and OVCAR-3 cells were sub-cloned by the clonal dilution method; one clone with a high expression of Wnt5A was chosen for SKOV-3 cells (clone C9 referred further as C9/SKOV-3 clone) and two clones from Wnt5A overexpressing OVCAR-3 cells were chosen (referred further as C2/OVCAR-3 clone and C3/OVCAR-3 clone) for further experiments. The expression of V5-tag was assessed by using an anti-V5 antibody (1/5000, Abcam, UK). C9/SKOV-3 clone, C3/OVCAR-3 clone, and C2/OVCAR-3 clone were transfected with 25 nM siRNA (ON-TARGET plus SMARTpool human Wnt5A, Cat# 1349–4176, Fisher Scientific AG, Wohlen, Switzerland), or 25 nM non-target siRNAs (ON-TARGETplus SMARTpool human NonTarget siRNA, Cat# 1153–7240, Fisher Scientific AG) known as scramble (Scr) as previously described^56^.Wnt5A expression at mRNA and protein level was detected as previously described^56^. Quantification of gene expression was performed via the standard curve method using REST-RG software version 3.

### Cell proliferation and colony-forming assay

C9/SKOV-3 clone, C3/OVCAR-3 clone, and mock control (7.5 × 10^3^) cells were seeded in a 96-well plate. Trypan blue was used to assess cell proliferation during 4–5 days after plating in the presence or absence of Wnt5A/FZD-5 antagonist Box5, a Wnt5a-derived N-butyloxycarbonyl hexapeptide (Met-Asp-Gly-Cys-Glu-Leu; 0.766 kDa, Tocris Bioscience, Bristol, UK) or CWHM-12 (MedChemExpress, NJ, USA) a specific inhibitor of αv integrin. For colony assay, C9/SKOV-3 clone, C3/OVCAR-3 clone, C2/OVCAR-3 clone, and mock control (300 cells/well) were plated in six-well plates in complete medium and allowed to grow for 11 days; colonies were assessed as previously described^20^.

### Scratch wound-healing assay and transwell invasion assay

Cells reached 90% confluency were starved overnight, then treated for 2 h with 10 µg/ml Mitomycin (Merck KGaA, Darmstadt, Germany). The wound was made by scratching the cell monolayer with a yellow tip and controlled after 3, 6, 12, 24 h with or without Box5 or in the presence or absence of CWHM-12. The invasiveness of Wnt5A overexpressing clones and mock cells was assessed (Calbiochem, USA) using 8 µm pore size transwells coated with Matrigel as previously described^56^.

### Spheroid formation assay

To assess the spheroid formation ability of C9/SKOV-3 clone versus mock 1.5 × 10^4^ cells were cultured in 6-well plates coated with 1% low melt agarose (IBI SCIENTIFIC, Tryon, NC, USA) in serum-free RPMI supplemented with 20 ng/ml recombinant human epithelial growth factor and 20 ng/ml recombinant human basic fibroblast growth factor (rhEGF, rhFGF, Royan Institute, Tehran, Iran) which were added every 48 h. After 6 days, the numbers of spheres greater than 70 μm in diameter were counted using an inverted microscope at 400× magnification.

### Flow cytometry assay

The cell cycle assay was performed using propidium iodide (PI) DNA staining. For that purpose, 1 × 10^6^ of mock, C9/SKOV-3 clone, and C3/OVCAR-3 clone cells were washed with PBS and fixed in 70% ethanol for 2 h at 4 ℃, washed again, pelleted by centrifugation, and stained with propidium iodide (50 µg/ml) in the presence of RNase A (100 µg/ml) (Sigma-Aldrich, Germany) for 15 min at 37 °C. Stained cells were analyzed using fluorescence-activated cell sorting BD FACS Calibur machine (San Jose CA, USA).

### Adhesion assay

A 96 well plate was coated with type -I or –IV collagen (10 µg/cm2) (Advanced BioMatrix, Inc), laminin (2 µg/cm2) (Sigma, Saint Louis, USA), fibronectin (5 µg/cm2) (Sigma, Saint-Louis, USA); non-specific binding sites were blocked by incubation with 2% bovine serum albumin (BSA) (Sigma-Aldrich, Saint-Louis, USA) for 2 h at room temperature (RT) in PBS. Then the adhesion assay was performed as previously described^20^.

### Integrin array assay

Assessment of integrin proteins on the cell surface of C9/SKOV-3 clone and mock in both monolayer and MCAs was performed by using the Alpha/Beta Integrin-Mediated Cell Adhesion Array Combo Kit (Chemicon, Billerica, Massachusetts, USA) according to the manufacture protocol as previously described^57^.

### Immunofluorescence and western blot analysis

pTyr^397^-FAK and E-cadherin (1:200, Santa Cruz Biotechnology, INC.) were detected in C9/SKOV-3 clone and mock cells using polyclonal rabbit anti-human pTyr^397^-FAK and polyclonal rabbit anti-human E-cadherin clone H-108 antibodies, respectively. Immunofluorescence was performed as previously described^19^. For detection of pTyr^397^-FAK cells were seeded into 96-well plate-coated with FN or LN and immunostained after 30 or 60 min. Staining was detected using goat anti-rabbit IgG Alexa Fluor-488 (1 µg/ml, Invitrogen, USA) or goat anti-mouse IgG Alexa Flour-568 (2 µg/ml, Invitrogen, USA) for 1 h at 37˚C. Nuclei were counterstained with DAPI (1 μg/ml, Invitrogen, USA). Western blot analysis was performed as previously described^18,20^ with the following antibodies: monoclonal mouse anti-human Wnt5A (1:1500, Abcam, Cambridge, UK), monoclonal mouse anti-human E-cadherin (1:1000, Santa Cruz Biotechnology, INC) and polyclonal rabbit anti-human GAPDH (1:1000, Abcam, Cambridge, UK) as a loading control.

### Human ovarian specimens

Serous type epithelial ovarian cancer (EOC) tumors and normal ovarian tissue specimens were obtained from surgeries performed at Imam- Khomeini University Hospital Complex. Approval was obtained from the institutional Ethics Committee on Human Investigation (Imam- Khomeini University Hospital Complex) following the World Medical Association guidelines (Helsinki Declaration of 2008) for research on human beings and informed consents were obtained from patients. All samples (n = 57) were examined by two independent and experienced gynecological pathologists for histological diagnosis and grade. The characteristics of patients are described in Supplementary Table S1. The patients (age = 24–71 and Median = 43) were divided into four groups: normal ovary (n = 10); borderline serous ovarian cancer (BLSOC, n = 12); low-grade serous ovarian cancer (LGSOC, n = 12) and high-grade serous ovarian cancer (HGSOC, n = 23). Samples were chopped into small pieces of 50 mg with a surgical bladder and immediately snap-frozen in liquid nitrogen for further RT-qPCR analysis of Wnt5A and integrins as described previously^56^. The sequences of primers are listed in Supplementary Table S2. The expression levels of target genes are normalized related to the ACTB gene. Quantification of gene expression was performed via the standard curve method using REST-RG software version 3.

### Hierarchical clustering analysis and functional and pathway enrichment analysis

A bidirectional hierarchical clustering heatmap of assessed genes in human specimens was constructed using gplots package of R language after extracting the expression values from the gene expression profile. Terms with a *P*-value of < 0.05 were collected and grouped into clusters based on their membership similarities. More specifically, *P*-values were calculated based on the cumulative hypergeometric distribution. The most significant term within a cluster was selected as the one representing the cluster. Subsequently, functional enrichment analysis was performed in 3 categories of GO terms: Biological process (BP), and Kyoto Encyclopedia of Genes and Genomes (KEGG) pathway enrichment were performed to determine the involvement of genes in different biological pathways by using Enrichr (a web-based enrichment analysis tool for non-ranked gene lists that is based on Fisher’s exact test). Moreover, protein–protein interaction between Wnt5A and integrins were analyzed with STRING v.11 (https://string-db.org/). GO terms with a *P*-value of < 0.05 were considered statistically significant and annotation results for GO (BP) were displayed using VENNY v2.1.

### Statistical analysis

The normality of nominal variables was analyzed by performing the Kolmogorov–Smirnov test. Skewed and normal distributed metric variables were analyzed between two groups using Mann– Whitney U or among multiple groups using Kruskal–Wallis and one-way ANOVA tests, respectively by using R version 3.5.2. Correlations between gene expressions were analyzed by the spearman's correlation coefficient test. All experiments were performed at least three times in triplicate and the results were expressed as mean + /- SD. P < 0.05 considered significant.

### Ethical approval

The study protocol followed the ethical guidelines of the Declaration of Helsinki Principles. The institutional review board of the University of Tehran and Royan institute approved this study (No. 961009257).

### Informed consent

An informed consent was obtained from all participants and/or their legal guardian.

## Supplementary Information

Supplementary Information

## Data Availability

All data generated or analyzed during this study are included in this published article and its Supplementary Information Files.
