# Supplementary material for: Wnt5A modulates integrin expression in a receptor-dependent manner in ovarian cancer cells
Source: Sci Rep. 2021 Mar 15;11:5885. doi: 10.1038/s41598-021-85356-6 (PMC7970989; doi:10.1038/s41598-021-85356-6)
Supplement: Supplementary file 1 — Supplementary Information [file 41598_2021_85356_MOESM1_ESM.pdf]

Wnt5A modulates integrin expression in a receptor-dependent manner in ovarian cancer cells

Vajihe Azimian-Zavareh[1,2], Zeinab Dehghani-Ghobadi[1], Marzieh Ebrahimi[3]*, Kian Mirzazadeh[1], Irina Nazarenko[4], Ghamartaj Hossein[1,4]*

1. Department of Animal Biology, School of Biology, College of Science, University of Tehran, Tehran, Iran.

2. Applied Physiology Research Center, Cardiovascular Research Institute, Isfahan University of Medical Sciences, Isfahan, Iran.

3. Department of Stem Cells and Developmental Biology, Cell Science Research Center, Royan Institute for Stem Cell Biology and Technology, ACECR, Tehran, Iran.

4. Institute for Infection Prevention and Hospital Epidemiology; Medical Center - University of Freiburg, Faculty of Medicine, University of Freiburg, 79106 Freiburg, Germany.

ghossein@ut.ac.ir; mebrahimi@royaninstitute.org

Table 1 Clinicopathological characteristics of patients

Tumor grade	Number of samples	Median age	Treatment	Time of sample collection (after diagnosis) Patient
Normal ovary	10	53	–	–
BLSOC[a]	12	42	None	AD[d]
LGSOC[b] (Grade I)	7	55	None	AD
LGSOC (GradeII)	5	47	None	AD
HGSOC[c] (Grade III)	12	56	None	AD
HGSOC (Grade lV)	12	61	None	AD

a: BLSOC (Borderline serous ovarian cancer), b: LGSOC (low-grade serous ovarian cancer), c: HGSOC (high-grade serous ovarian cancer), d: AD (After diagnosis, before treatment).

Table S2: Primer sequences used for RT-qPCR

Primer name	Sequence
GAPDH	F: 5´GAAATCCCATCACCATCTTCC3´<br>R: 5´GGCTGTTGTCATACTTCTCAT3´
ACTB	F: 5´CTTCCTTCCTGGGCATG-3'<br>R: 5´GTCTTTGCGGATGTCCAC-3'
CDH-1	F: 5´GCTCTCCACTCTTACTTCCT3´<br>R: 5´GTTTGGTCTGATGCG3´
CDH-2	F: 5´GCCCAAGACAAAGAGACCC3´<br>R: 5´CTGCTGACTCCTTCACTGAC3´
EpCAM	F: 5´CCATGTGCTGGTGTGTGAAC-3'<br>R: 5´CCTTCTGAAGTGCAGTCCGC-3'
WNT5A	F: 5'- GCCATGAAGAAGTCCATTG -3'<br>R: 5'- AGCGACCACCAAGAATTG-3'
ITGB1	F: 5′- GTGGGTGGTGCACAAATTC-3′<br>R: 5′-GGTCAATGGGATAGTCTTCAGC-3′
ITGB2	F: 5′-TTCGGGTCCTTCGTGGACA-3'<br>R: 5′- ACTGGTTGGAGTTGTTGGTCA-3'
ITGB3	F: 5′- AGCCAACAACCCACTGTA-3'<br>R: 5′- CTGACATTCTCCCAACCTAC-3'
ITGB4	F: 5′-TGGAAGTACTGTGCCTGCTG-3'<br>R: 5′-TGCATGTTGTTGGTGACCTT-3'
ITGB6	F: 5′- TCCATCTGGAGTTGGCGAAAG-3'<br>R: 5′- TCTGTCTGCCTACACTGAGAG-3'
ITGA4	F: 5′- AGCCCTAATGGAGAACCTTGT-3'<br>R: 5′- CCAGTGGGGAGCTTATTTTCAT-3'
ITGA5	F: 5'- TTTATCGGTCTCGGGAGTTG-3'<br>R: 5'-CTTCAACTTAGACGCGGAGG -3'
ITGAV	F: 5'- GCAACAGGCAATAGAGAT-3'<br>R: 5'- TGCTGAATCCTCCTTGACAA-3'
ROR-2	F: 5'- AATCACAGCGGCCTTCACC-3'<br>R: 5'- GGCACAGGTCGCTCTCCA-3'
ROR-1	F: 5'- TAATCGGAGAGCAACTTCA-3'<br>R: 5'- TGTAGTAATCAGCGGAGTAA-3'
FZD-2	F: 5'- CGTCCTCAAGGTGCCATCC-3'<br>R: 5'- GCAGCCCGACAGAAAAATGA-3'
FZD-4	F: 5'- CCTCGGCTACAACGTGACC-3'<br>R: 5'- TGCACATTGGCACATAAACAGA-3'
FZD-5	F: 5'- TTCTGGATAGGCCTGTGGTC-3'<br>R: 5'- CGTAGTGGATGTGGTTGTGC-3'

Fig. S1

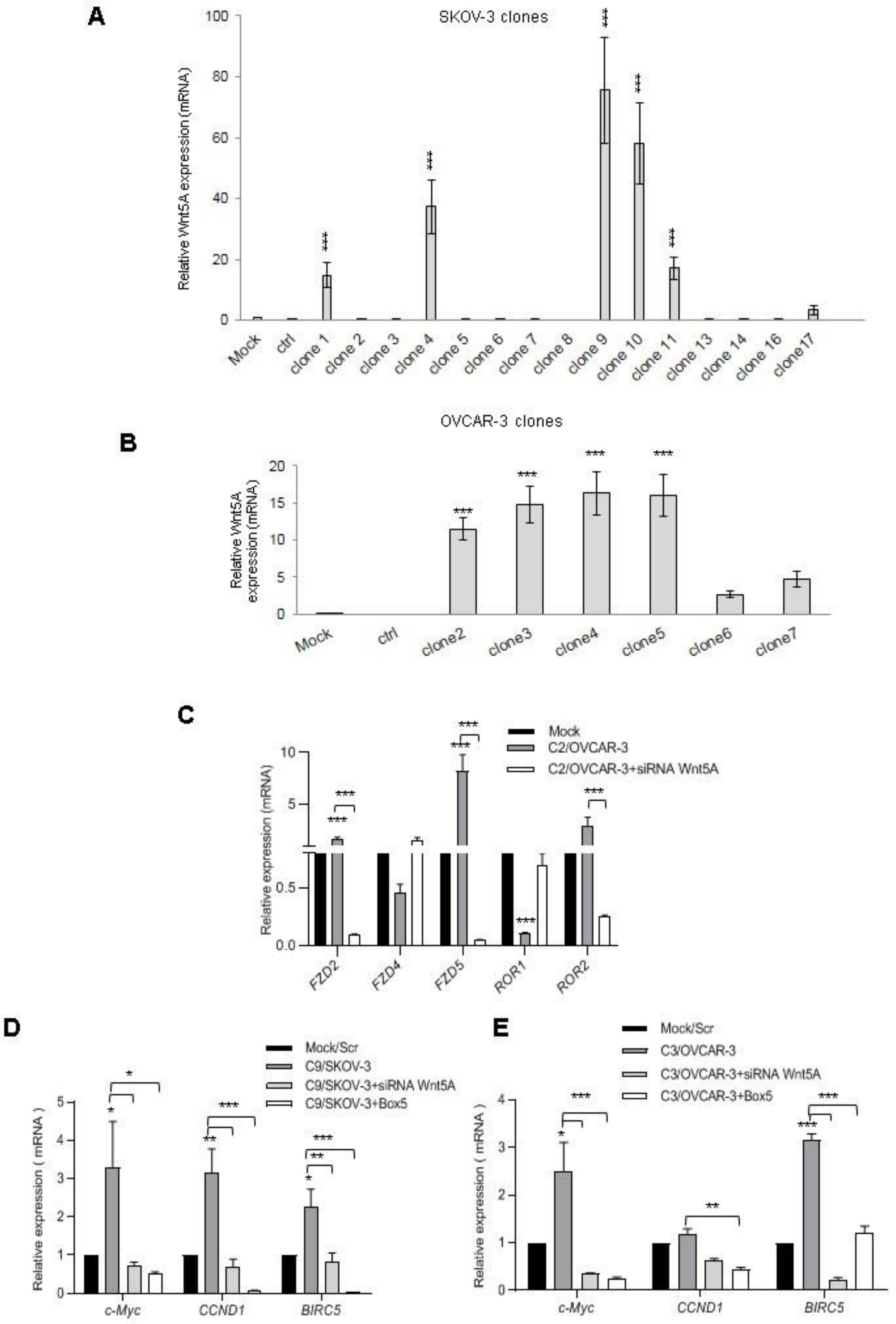
A
SKOV-3 clones
Relative Wnt5A expression (mRNA)
0
20
40
60
80
100
***
Mock
ctrl
clone 1
clone 2
clone 3
clone 4
clone 5
clone 6
clone 7
clone 8
clone 9
clone 10
clone 11
clone 13
clone 14
clone 16
clone17
B
OVCAR-3 clones
Relative Wnt5A expression (mRNA)
0
5
10
15
20
***
Mock
ctrl
clone2
clone3
clone4
clone5
clone6
clone7
C
Mock
C2/OVCAR-3
C2/OVCAR-3+siRNA Wnt5A
Relative expression (mRNA)
0.0
0.5
5
10
***
FZD2
FZD4
FZD5
ROR1
ROR2
D
Mock/Scr
C9/SKOV-3
C9/SKOV-3+siRNA Wnt5A
C9/SKOV-3+Box5
Relative expression (mRNA)
0
1
2
3
4
5
*
**
***
c-Myc
CCND1
BIRC5
E
Mock/Scr
C3/OVCAR-3
C3/OVCAR-3+siRNA Wnt5A
C3/OVCAR-3+Box5
Relative expression (mRNA)
0
1
2
3
4
*
**
***
c-Myc
CCND1
BIRC5

Fig. S1 Wnt5A modulates its own receptors and cell cycle markers. (A, B) Sub-cloning of Wnt5A overexpressing SKOV-3 and OVCAR-3 cells. Wnt5A stably transfected SKOV-3 and OVCAR-3 cells (upper and lower panel) were sub-cloned by clonal dilution method and Wnt5A expression level was assessed by RT-qPCR. Results were normalized relative to GAPDH level as an internal control (means ± SD; n=3). **(C)** RT-qPCR analysis of frizzled-2 (FZD-2), frizzled-4 (FZD-4), frizzled-5 (FZD-5), ROR-1, and ROR-2 in C2/OVCAR-3 clone with or without siRNA Wnt5A transfection, relative to mock or scrambled (scr). GAPDH was used as an internal control. **(D, E)** Increased levels of c-myc, CCND1 (cyclin D1), and BIRC5 (Survivin) mRNA levels in C9/SKOV-3 and C3/OVCAR-3 clones compared to mock which was abrogated in Wnt5A knock-down or Box5-treated clones. Results of RT-qPCR were normalized related to GAPDH used as an internal control. Mean ± SD of at least three independent experiments. *: $P < .05$; **: $P < .01$; ***: $P < .001$ compared to mock.

Fig. S2

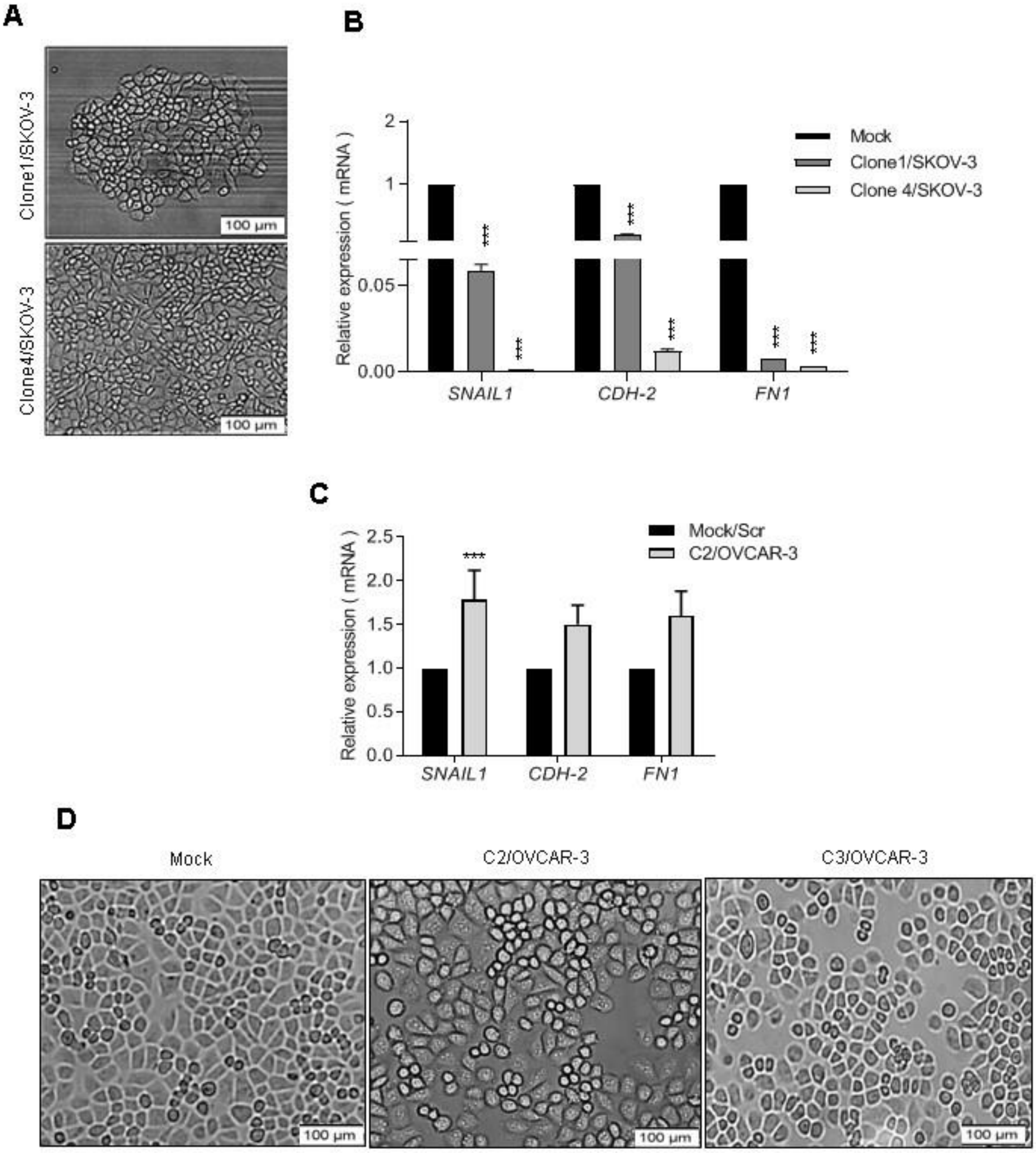

Fig. S2 Wnt5A affects the morphology of the C9/SKOV-3 clone and modulates the expression levels of mesenchymal markers. (A) Clone 1 and clone 4 cells showed epithelial-like morphology compared to spindle morphology of non-transfected cells (scale bar: 100 µm). **(B)** Clone 1 and clone 4 cells showed decreased mRNA levels of CDH-2 (N-cadherin), SNAIL (Snail), and FN1 (fibronectin) expression levels as mesenchymal markers compared to mock.

(C) C2/OVCAR-3 clones showed increased mRNA levels of CDH-2 (N-cadherin), SNAIL (Snail), and FN1 (fibronectin) expression levels as mesenchymal markers. **(D)** Epithelial-like-morphology of C2/OVCAR-3 and C3/OVCAR-3 clones was not changed relative to mock cells. Results of RT-qPCR were normalized related to GAPDH used as an internal control. Mean ± SD of at least three independent experiments. *: P <.05; **: P <.01 ***; P <.001 relative to mock.

Fig. S3

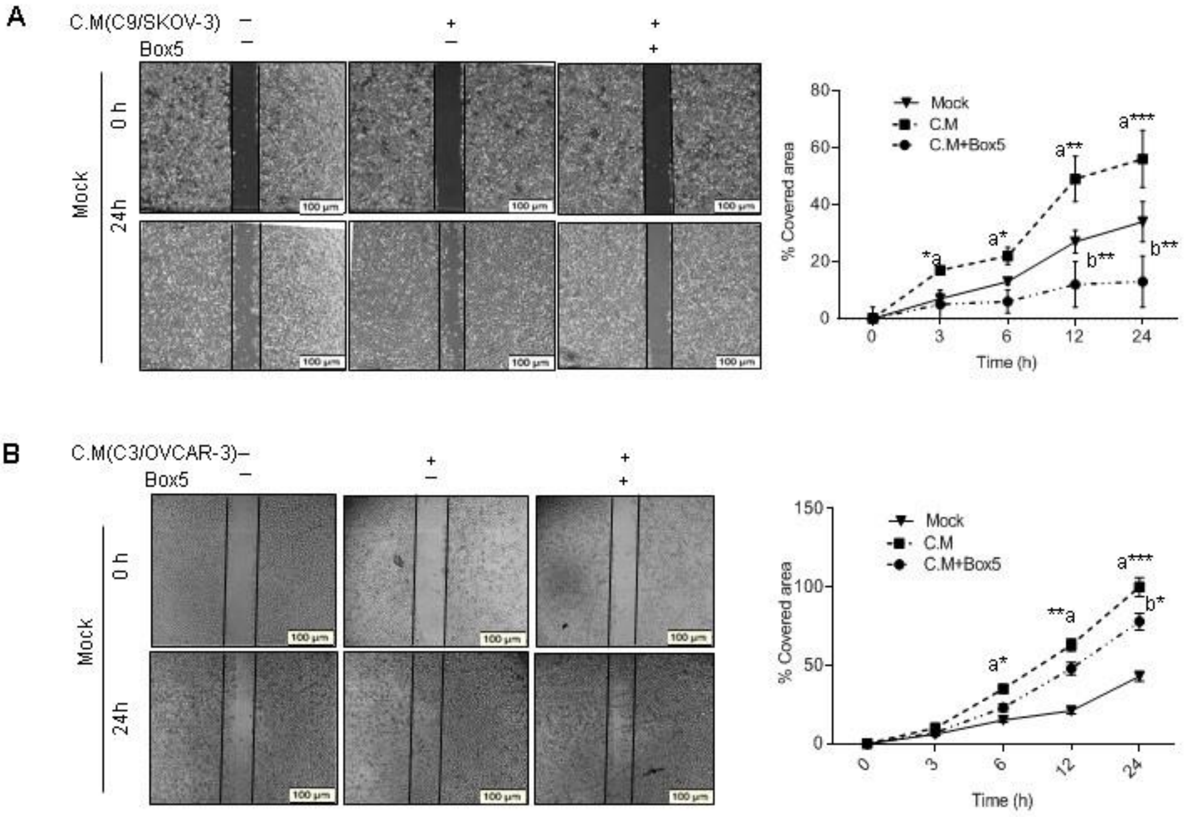

Fig. S3 Condition medium of Wnt5A overexpressing clones affects migration of SKOV-3 and OVCAR-3 mock cells.

(A, B) Wound-healing analysis of mock cells during a 24-h time course in the presence of C9/SKOV-3 and C3/OVCAR-3-derived conditioned medium (C.M) with or without Box5 (1000 and 250 µM, respectively). Percent of wound closure was determined in different time points 0, 3, 6, 12, and 24h after scratching. Motility of mock cells was increased in the presence of condition medium (C.M) of C9/Wnt5A and C3/OVCAR-3 clones which was abrogated upon addition of Box5 (n=3, mean ± SD; scale bar: 100 µm). a: compared to mock, and b: compared to condition medium (C.M) alone. *: P <.05; **: P <.01; ***: P <.001

Fig. S4

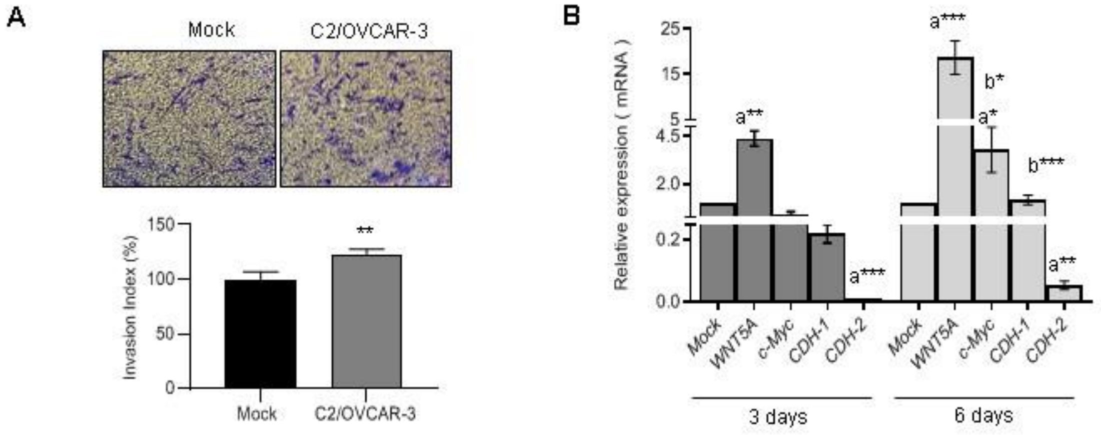

Fig. S4 Wnt5A overexpression affects invasion and mesenchymal markers. (A) C2/OVCAR-3 clone showed increased cell invasion compared to mock (Upper panel, photos are representative of one of three performed experiments). The lower panel shows quantification of cell invasion by counting cells at ten random fields (Scale bar: 100 µm). **(B)** mRNA levels of Wnt5A, CDH-1, CDH-2, and c-myc were assessed in 3 and 6 days C9/SKOV-3 and mock MCAs showed increased Wnt5A, c-myc, and CDH-1 at 6 days of culture and decreased levels of CDH-2. Results of RT-qPCR were normalized related to GAPDH used as an internal control. Mean ± SD or at least three independent experiments. *: P <.05; **: P <.01 ***; P <.001. a: relative to mock, and b: relative to 3 days MCAs.

Fig. S5

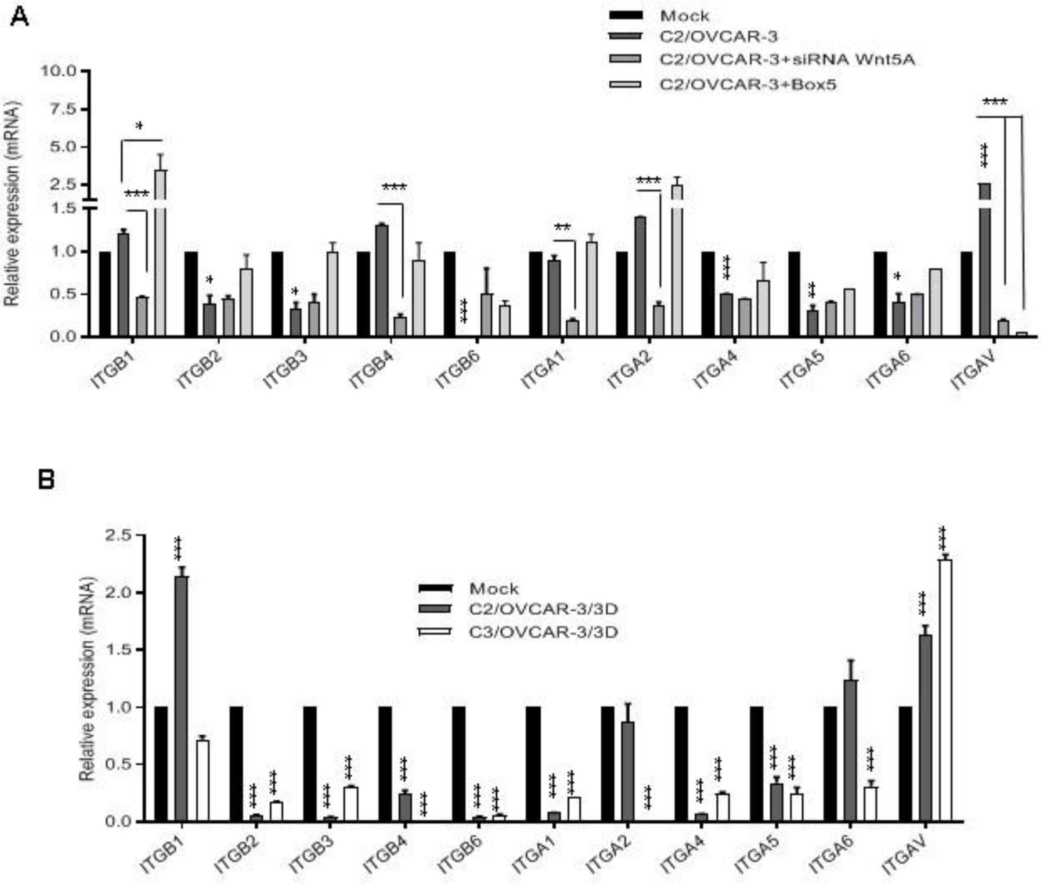

Fig. S5 Wnt5A overexpression affects integrin expression in 2D and 3D models. RT-qPCR analysis of ITGB1, ITGB2, ITGB3, ITGB4, ITGB6, ITGA1, ITGA2, ITGA4, ITGA5, ITGAV, and ITGA6 integrins. **(A)** The monolayer of C2/OVCAR-3 in the presence or absence of siRNA Wnt5A transfection or Box5, and **(B)** C2/OVCAR-3 and C3/OVCAR-3 MCAs compared to mock. Results of RT-qPCR were normalized related to GAPDH used as an internal control. Mean ± SD; n=3 *: P <.05; **: P <.01 ***; P <.001 relative to mock or scrambled (scr). MCAs: multicellular aggregates.

Fig. S6

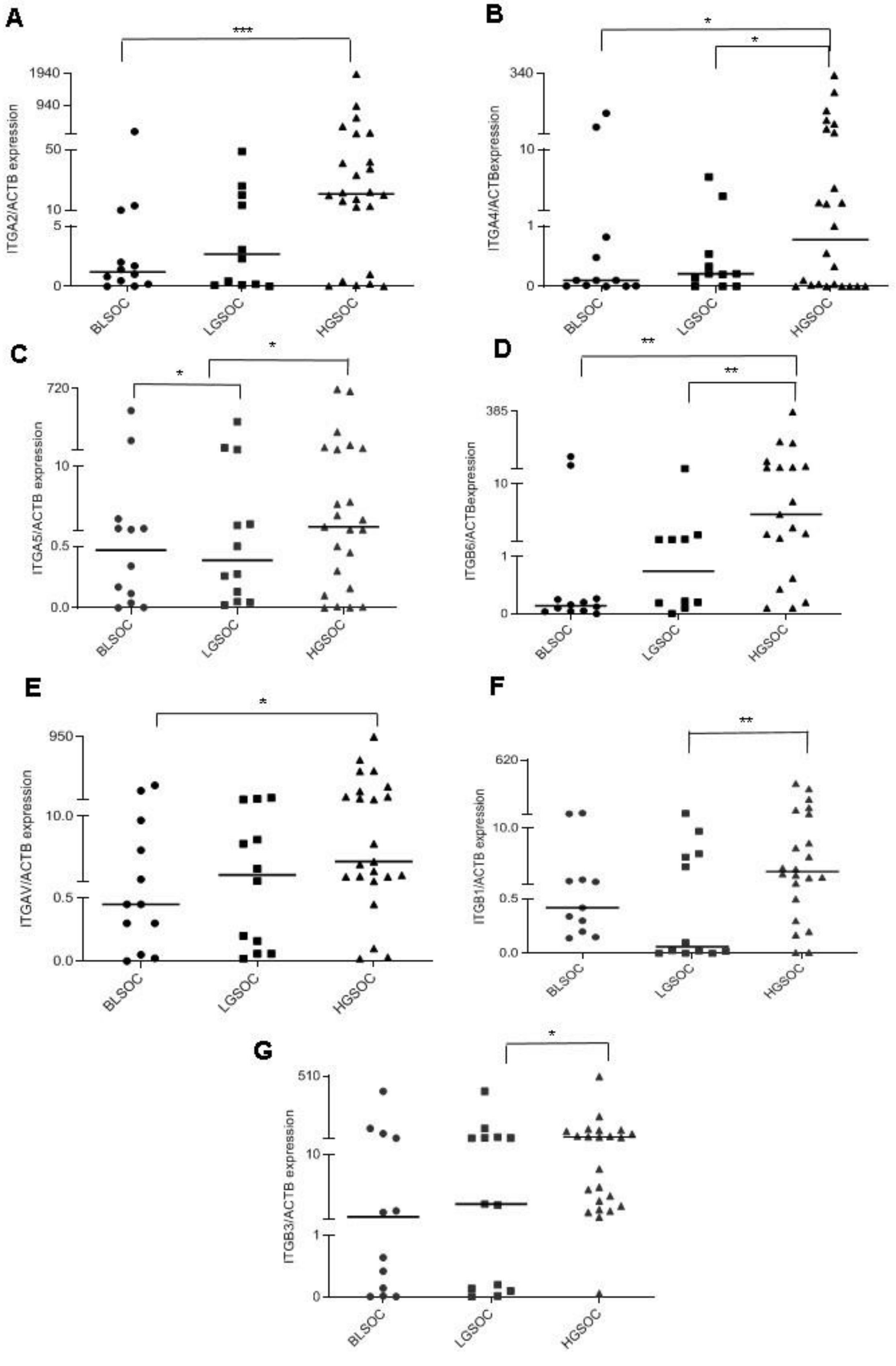

A
***
1940
940
50
10
5
0
ITGA2/ACTB expression
BLSOC
LGSOC
HGSOC
B
*
*
340
10
1
0
ITGA4/ACTBexpression
BLSOC
LGSOC
HGSOC
C
*
*
720
10
0.5
0.0
ITGA5/ACTB expression
BLSOC
LGSOC
HGSOC
D
**
**
385
10
1
0
ITGB6/ACTBexpression
BLSOC
LGSOC
HGSOC
E
*
950
10.0
0.5
0.0
ITGAV/ACTB expression
BLSOC
LGSOC
HGSOC
F
**
620
10.0
0.5
0.0
ITGB1/ACTB expression
BLSOC
LGSOC
HGSOC
G
*
510
10
1
0
ITGB3/ACTB expression
BLSOC
LGSOC
HGSOC

Fig. S6 Differential expression levels of integrin subunits in BLSOC, LGSOC and HGSOC human specimens. The extracted RNA from fresh serous type EOC tumors (n = 47) were divided into three groups borderline serous ovarian cancer (BLSOC, n=12); low-grade serous ovarian cancer (LGSOC, n=12) and high-grade serous ovarian cancer (HGSOC, n=23), as well as fresh normal ovarian tissue (n = 10), were analyzed by RT-qPCR for the following mRNA expression levels: **(A)** ITGA2, **(B)** ITGA4, **(C)** ITGA5, **(D)** ITGB6, **(E)** ITGAV, **(F)** ITGB1 and **(G)** ITGB3. Dot plots show the distribution of normalized RT-qPCR analysis for afore-mentioned genes expression levels in tumor specimens related to normal healthy ovaries. Values were normalized relative to ACTB expression levels used as an internal control. *: P <.05; **: P <.01; ***: P <.001 compared to normal ovary.
